# DNA methylation signatures link prenatal famine exposure to growth and metabolism

**DOI:** 10.1038/ncomms6592

**Published:** 2014-11-26

**Authors:** Elmar W. Tobi, Jelle J. Goeman, Ramin Monajemi, Hongcang Gu, Hein Putter, Yanju Zhang, Roderick C. Slieker, Arthur P. Stok, Peter E. Thijssen, Fabian Müller, Erik W. van Zwet, Christoph Bock, Alexander Meissner, L. H. Lumey, P. Eline Slagboom, Bastiaan T. Heijmans

**Affiliations:** 1 Molecular Epidemiology, Leiden University Medical Center, 2300RC Leiden, The Netherlands; 2 Medical Statistics and Bioinformatics, Leiden University Medical Center, 2300RC Leiden, The Netherlands; 3 The Broad Institute of MIT and Harvard, Cambridge, Massachusetts 02142, USA; 4 Human Genetics, Leiden University Medical Center, 2300RC Leiden, The Netherlands; 5 Computational Biology and Applied Algorithmics, Max Planck Institute for Informatics, 66123 Saarbrücken, Germany; 6 CeMM Research Center for Molecular Medicine, Austrian Academy of Sciences, 1090 Vienna, Austria; 7 Department of Laboratory Medicine, Medical University of Vienna, 1090 Vienna, Austria; 8 Department of Stem cell and Regenerative Biology, Harvard University, Cambridge, Massachusetts 02138, USA; 9 Department of Epidemiology, Mailman School of Public Health, Columbia University, New York, New York 10032, USA

## Abstract

Periconceptional diet may persistently influence DNA methylation levels with phenotypic consequences. However, a comprehensive assessment of the characteristics of prenatal malnutrition-associated differentially methylated regions (P-DMRs) is lacking in humans. Here we report on a genome-scale analysis of differential DNA methylation in whole blood after periconceptional exposure to famine during the Dutch Hunger Winter. We show that P-DMRs preferentially occur at regulatory regions, are characterized by intermediate levels of DNA methylation and map to genes enriched for differential expression during early development. Validation and further exploratory analysis of six P-DMRs highlight the critical role of gestational timing. Interestingly, differential methylation of the P-DMRs extends along pathways related to growth and metabolism. P-DMRs located in *INSR* and *CPT1A* have enhancer activity *in vitro* and differential methylation is associated with birth weight and serum LDL cholesterol. Epigenetic modulation of pathways by prenatal malnutrition may promote an adverse metabolic phenotype in later life.

## Abstract (web summary)

The long-term effect of prenatal nutrition on gene regulation is largely unknown. Here the authors identify differentially methylated regions in whole blood from individuals exposed to famine early after conception, and show that these epigenetic changes may have adverse metabolic consequences later in life.

## Graphical abstract

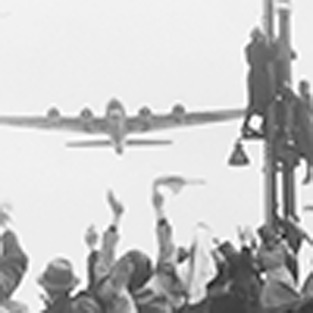

Epigenetic regulation of gene expression can undergo persistent changes as a result of environmental exposures, but the contribution of this mechanism to human disease remains to be defined^1^. Animal models exposed to malnutrition during prenatal development show epigenetic changes at the level of DNA methylation in specific promoters^2^, retrotransposons^3^ and imprinted regions^4^ that explain phenotypic variation in adult animals. Few study designs can address the question whether these relationships also occur in humans. These include studies on well-documented historical famines that provide a quasi-experimental setting with a long follow-up. Prenatal exposure to the Dutch Hunger Winter, a severe war-time famine at the end of World War II, is associated with an adverse metabolic profile (suboptimal glucose handling, higher body mass index (BMI), elevated total and low-density lipoprotein (LDL) cholesterol) and a higher risk of schizophrenia in later life^5^. The subsequent observation of differential DNA methylation after prenatal famine exposure at promoters and imprinted regions regulating genes involved in metabolism suggested a role for epigenetic mechanisms in these phenotypic associations^6^,^7^. These findings may have a broader significance since similar health outcomes and DNA methylation differences were reported for common adverse prenatal conditions, including exposure to gestational diabetes^8^,^9^ and maternal smoking during pregnancy^10^,^11^.

As of yet, comprehensive genome-scale views of differential methylation following prenatal (mal)nutrition are lacking in humans. The characterization of the genomic regions and biological pathways involved will be key to understand the environmentally induced plasticity of the epigenome and its asserted role in disease^12^. Here we use reduced representation bisulfite sequencing (RRBS)^13^ to generate DNA methylation data in whole blood on 1.2M individual CpG dinucleotides in 24 individuals prenatally exposed to famine and 24 unexposed same-sex sibling as controls. We focus on exposure in early gestation since this developmental period represents a window of increased sensitivity^7^,^14^ and extensive epigenetic reprogramming^15^. Using a step-wise analysis strategy based on extensive genomic annotation^16^, followed by technical and biological validation of selected individual regions, we identify genomic characteristics of prenatal malnutrition-associated differentially methylated regions (P-DMRs). Moreover, we identify pathways accumulating P-DMRs and report individual P-DMRs that showed enhancer activity *in vitro* and were tentatively associated with phenotypic outcomes related to early gestational famine exposure. In this study, we encounter and address most issues inherent to epigenetic epidemiology^1^ and present an approach for the use of bisulfite sequencing data in an epidemiological setting.

## Results

### Exposure and subject selection for bisulfite sequencing

The Hunger Winter was the consequence of a German-imposed food embargo in the western part of The Netherlands and lasted from November 1944 to May 1945. Official daily rations were set weekly during the famine period and were the same for every individual. The daily rations fell below 1,000 kcal (mean=667 kcal (s.d., 151)), and there was little variation in the percentage of calories from proteins (12%, of which 4% of animal origin), fat (19%) and carbohydrates (69%)^17^. During the famine period, registries and health care remained intact, so that individuals who were prenatally exposed to this famine can be traced from birth records and the timing of the exposure can be established^18^. Sixty individuals were available for epigenetic analysis who were exposed to famine during early gestation (from conception up to 10 weeks into development) and who could be compared with a prenatally unexposed same-sex sibling to control for genetic and familial background. For the genome-scale RRBS measurements, we focused on a subset of 24 sibling pairs (Supplementary Fig. 1A.1) with a <5-year age difference and an equal number of male and female pairs as well as an equal number of pairs with the control siblings conceived and born before or after the famine period to minimize the potential effects of these possible confounders (Supplementary Table 1).

### Genome-scale analysis

We employed RRBS on DNA from whole blood to obtain single-nucleotide high-resolution DNA methylation data on a genomic scale^13^. Sequencing on an Illumina GAIIx, utilizing one sequencing lane per individual (*N*=48), resulted in an average of 25.6 million high-quality reads per sample (Supplementary Table 1). We excluded differences in overall methylation between the exposed and their siblings, both for all high-quality reads (*P*=0.91) and for all repetitive elements^19^, including *LINES1* which are often measured as a proxy for global methylation (*P*_FDR_>0.26; subset of *LINES*1: *P*_FDR_>0.77, Supplementary Fig. 1A.2).

**Figure 1:**
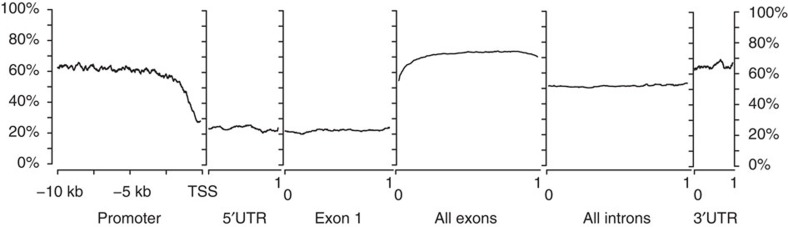
The methylation level of genes in the RRBS data set. The methylation level across a gene. A lowess has been fit across all data for all entrez genes. The width of the gene elements represents the relative amount of data for the elements in the total data set.

After aligning the reads to the non-repetitive genome, we excluded CpG sites with a low or extremely high coverage (median <6 or >200) and those that were uninformative (mapping to a random chromosome, median methylation of 0 or 100%). This resulted in 1,206,161 unique CpG sites with an average median sequencing depth of 28-fold and an average data completeness per CpG site of 99.8% (Supplementary Table 2, Supplementary Fig. 2). Among the 1.2M CpG dinucleotides measured, regions within and near genes were somewhat over-represented as compared with the genome (Supplementary Fig. 3). The 1.2M CpG dinucleotides showed the canonical bimodal distribution of DNA methylation (Supplementary Fig. 4, average methylation 61.2% (s.d. 1.7%)) also found by whole-genome bisulphite sequencing (WGBS)^20^. The promoter and intragenic methylation levels showed a similar pattern and levels as reported in blood monocytes using WGBS^20^ (Fig. 1).

Given the limited sample size of our study and the expected moderate effect sizes^7^,^21^, we were unable to test each CpG site individually; therefore, we evaluated these 1.2M CpG dinucleotides grouped by external genomic annotations, testing each individual genomic annotation for an overall association with early gestational famine exposure (Supplementary Fig. 1A.3). Testing genomic annotations instead of 1.2M individual CpG sites reduces the burden of multiple testing, increases statistical power and aids interpretation of results^16^, but restricted our analyses to CpG sites contained in such annotations, resulting in a less comprehensive genome-scale assessment than possible if our study numbers were larger. We selected 28 genomic annotations from the literature, including standard gene-based annotations (for example, promoters, CpG islands) and those relevant to development (for example, developmental enhancers, Table 1 and Methods). Although RRBS enriches for genomic regions with a higher GC content, relatively CpG-poor annotations were also represented. For five annotations, an overall association of prenatal famine with differential methylation was detected (*P*_FDR_<0.05; Table 1). These annotations included non-CGI promoters (‘bona fide non-CGI promoters’^22^), enhancers^23^, exons, DNaseI/FAIRE-seq open chromatin regions and enhancers active during the pre- and peri-implantation period^24^. These results thus highlight regions with a potential regulatory function. There was no difference between the average GC content of the five associated annotations and the non-significantly associated genomic annotations excluding technical bias during bisulfite sequencing as an explanation (*t*-test, *P*=0.79).

### Characteristics of DMRs associated with prenatal famine

**Figure 2:**
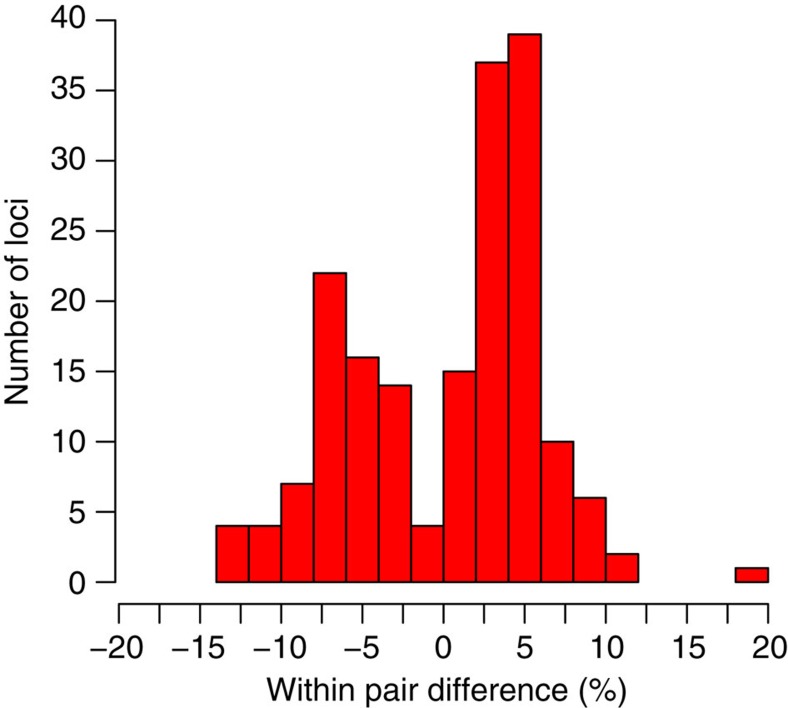
The average within-pair difference for the 181 regions associated with prenatal famine exposure after correction for multiple testing. A histogram for the average within pair difference (%) between the exposed and unexposed siblings. A positive number reflects relative higher DNA methylation levels in the exposed.

The five associated genomic annotations comprised of 90,451 regions covered by RRBS (with a median length of 1.4 kb and harbouring on average 6.7 CpG dinucleotides). The 90,451 regions were individually tested for an association with prenatal famine exposure (Supplementary Fig. 1A.4). This analysis identified 181 regions as prenatal-malnutrition associated differentially methylated regions (P-DMRs, *P*_FDR_<0.05, Supplementary Data 1). The difference in DNA methylation between exposed individuals and their same-sex sibling was variable at these regions (up to >10%), but generally moderate (median 4.6%; Fig. 2). Relative hypermethylation of P-DMRs among exposed individuals was more common (60.8%) than relative hypomethylation (39.2%). Interestingly, nutrient deficiency was recently implicated in reduced *Tet*-dependent demethylation during blastocyst development in mice^25^.

We then set out to characterize the P-DMRs (Supplementary Fig. 1A.5). Of the 181 P-DMRs, a majority of 60.7% was located in gene bodies, 11.6% in proximal upstream regions (−10 to 0 kb), 10.5% in distal upstream regions (−100 to 10 kb), 10.0% in downstream regions (0 to 100 kb) and 7.2% in regions not mapping to a gene (>100 kb). The relative high occurrence of gene bodies is in line with the large proportion of CpG dinucleotides mapping to gene bodies (51% of all CpGs in the human genome and 53% of CpGs interrogated with RRBS; Supplementary Fig. 3) as well as the frequent overlap with open chromatin, the largest contributor to the 90,451 regions, and both gene bodies and enhancers^26^. We then further characterized the P-DMRs by several enrichment tests, contrasting the 181 P-DMRs against the non-significant regions in the five associated annotations. First, we employed EpiGRAPH^27^, a software for testing the co-occurrence of genomic regions with particular histone marks in public data sets. The 181 P-DMRs showed an increased co-occurrence with histone marks associated with active enhancers, transcribed gene bodies, active regulatory sites and expressed exons in comparison with the non-significant regions in the five associated genomic annotations (H3K4me1-3, H2AZ, H3K9me1, PolII, H3K79me1, H3K27me1). Also, a lower co-occurrence with SINEs and a lower overall repeat score was observed (*P*_FDR_<0.05). Interestingly, although the putative P-DMRs were identified on the basis of whole blood samples, the genes nearest to the P-DMRs were not enriched for genes showing tissue-specific gene expression including an absence for enrichment for blood and bone marrow (odds ratio (OR)=0.98 (95% confidence interval (CI): 0.59–1.64) and OR=1.01 (95% CI: 0.55–1.87)). In line with these data, the majority of P-DMRs (*N*=128) did not overlap with tissue DMRs (tDMRs) identified in a comprehensive WGBS data set covering 35 fetal and somatic tissues^28^. In contrast, the P-DMRs were enriched for genes differentially expressed during the pre-implantation stage of development (OR=4.95 (95% CI: 3.53–6.49) (ref. 29)) and organogenesis (OR=4.76 (95% CI: 3.11–7.30) (ref. 30)) in humans. Thus, the set of P-DMRs may reflect the early gestational timing of the exposure rather than the investigated tissue.

### Validation of genome-scale measurements

**Figure 3:**
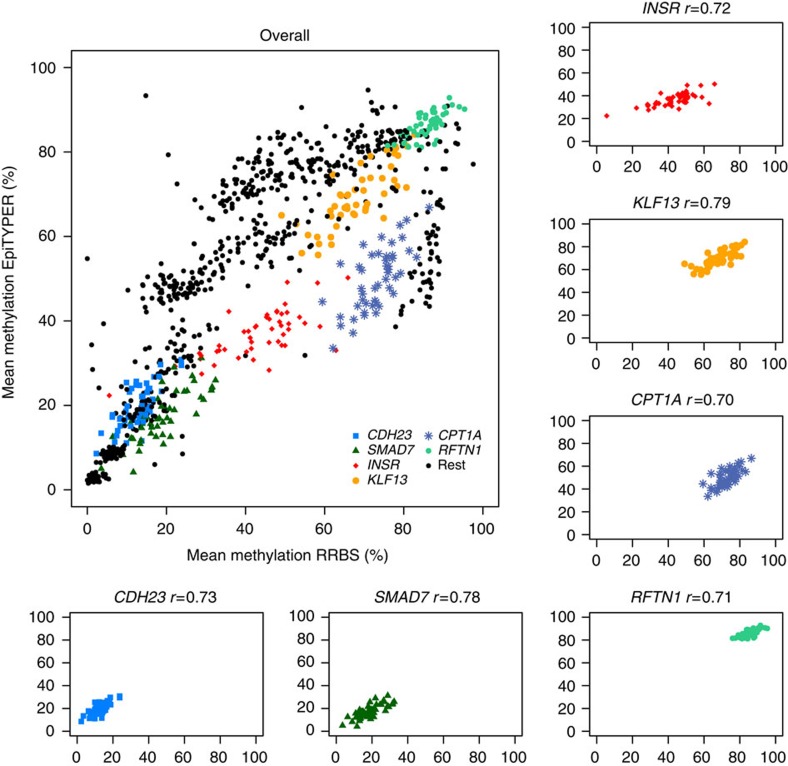
The correspondence between RRBS and EpiTYPER measurements of DNA methylation at P-DMRs. Individual regions with a Pearson correlation >0.7 are denoted in color and plotted separately along the main figure. The correlation of the other regions can be found in Supplementary Table 3.

Next, we went on to explore the characteristics of individual P-DMRs in more detail, starting with a technical validation of our findings in the same 48 individuals with the mass spectrometry-based method EpiTYPER^31^ (Supplementary Fig. 1A.6). From the 181 significant P-DMRs, we prioritized 11 regions with the lowest *P* values and nine significant regions according to consistency of DNA methylation differences across multiple CpG dinucleotides, and their mapping to genes with a known function. The prioritized P-DMRs had a similar distribution in DNA methylation levels to all 181 P-DMRs (Supplementary Table 3). For 19/20 regions, a working assay could be designed (Supplementary Table 4). The overall correlation between the average DNA methylation measured using the genome-wide method RRBS and the locus-specific method EpiTYPER was good (*r*=0.81; Fig. 3), despite the fact that the length of RRBS regions defined by genome annotations were larger (>1 kb) than the regions that could be targeted by EpiTYPER assays (<600 bp, Supplementary Table 5). Of the 19 regions, 13 were again associated with famine in the EpiTYPER data (*P*<0.05, Supplementary Table 3), although some of the effect sizes were attenuated as compared with those originally observed in the genome-scale data. This may be related to the difference in size of the region measured (EpiTYPER assays cannot target full RRBS regions) and the difference in accuracy of the DNA methylation estimates.

For subsequent analyses, we focused on P-DMRs for which the EpiTYPER assay provided a good representation of the larger RRBS region as defined by a Pearson’s correlation coefficient >0.7 between both measurements in the same individuals (Supplementary Fig. 1B.1). Six loci met this criterion and mapped to *CDH23*, *SMAD7*, *INSR*, *CPT1A*, *KLF13* and *RFTN1* (Fig. 3). These six EpiTYPER assays were subsequently measured in all available 60 individuals exposed during early gestation and their same-sex sibling controls available in the Hunger Winter Families Study, thus extending the analyses from 48 individuals to 120 individuals in total (Supplementary Fig. 1B.2).

DNA methylation differences in the 60 sibling pairs were consistent for CpG dinucleotides measured across the P-DMRs with the EpiTYPER assays (Supplementary Table 6), although attenuated as compared with those originally observed in the genome-scale data (Table 2). Next, we verified that the differential methylation at the P-DMRs was independent of the current, post-natal environment in these 60 sibling pairs. The associations with prenatal famine were not affected by age, smoking (neither current smoking nor package years), socio-economic status (SES), current diet (kcal per day nor the percentage of fat, carbohydrates or protein in the diet), BMI, known medical conditions and related medicine use. Finally, we excluded cellular heterogeneity of whole-blood samples as a confounding factor by exploring the potential association of DNA methylation at the six regions with blood cell counts in an independent sample set (Supplementary Table 7).

### Critical window of exposure

**Figure 4:**
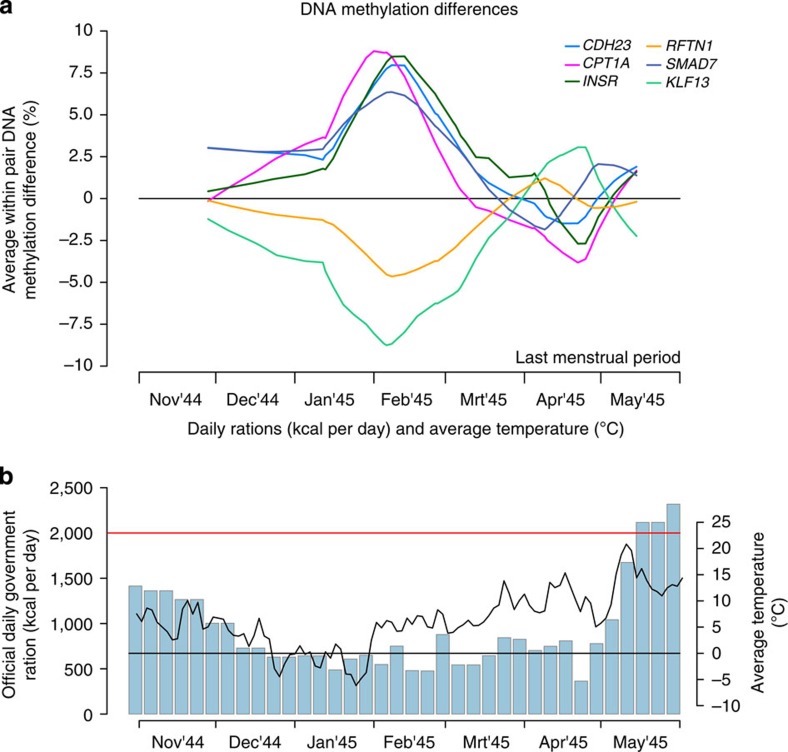
Results across the famine period. (**a**) A lowess curve depicting the average within-pair difference (*y* axis) stratified by the estimate of the start of pregnancy (LMP; *x* axis). Each coloured line represents an individual P-DMR. (**b**) the blue bars depict the official daily rations (kcal per day) per week, the black line represents a lowess curve depicting the average 24 h temperature (source KNMI; DeBilt weather station). The daily requirement of (non-pregnant) women of 2,000 kcal per day is denoted in red.

The influence of a prenatal exposure on DNA methylation critically depends on its exact timing during development^7^. The gestational timing of the exposure to famine was based on the mother’s last menstrual period (LMP), a commonly used proxy for the start of pregnancy. A significant interaction was observed between the start of pregnancy and famine exposure on DNA methylation for the six P-DMRs (*P*_interaction_=0.016), which is indicative of a critical period during early gestation. We further explored this observation by plotting the average within sibling pair difference in DNA methylation of the six P-DMRs against the start of pregnancy (Fig. 4a). DNA methylation differences were present throughout the first 4 months of the Famine, but waned towards the end to become virtually absent by the start of April 1945 (test for interaction pre-April and later pregnancies, *P*_interaction_=4.6 × 10^−3^). Re-examination of other loci identified as P-DMRs in earlier studies of the same individuals^6^,^7^,^32^, showed the same attenuation of DNA methylation differences in pregnancies starting after April 1945 (*P*_interaction_=1.8 × 10^−3^).

To evaluate the consistency of such a pre-April difference, we revisited the genome-scale RRBS and the EpiTYPER validation data for these six P-DMRs (Supplementary Fig. 1B.3). Inspection of the RRBS data in exposed individuals from pre-April pregnancies and sibling controls (36 individuals) showed that the DNA methylation differences for the six P-DMRs were virtually identical to those in the discovery set (48 individuals; Table 3). Moreover, these pre-April associations were reliably validated in the EpiTYPER validation data for the complete set of pre-April pregnancies (72 individuals). The effect sizes of associations for the six P-DMRs were remarkably similar for the pre-April sibling pairs from the discovery set and the siblings pairs used in the biological validation (Table 3). The replication rate of these associations was high (95% CI: 3/6–6/6). Conversely, no DNA methylation differences were observed in the EpiTYPER validation data at the six P-DMRs in sibling pairs comprising an exposed individual conceived later during the famine period (*P*>0.20). We did not observe evidence for sex-specific effects in pre-April pregnancies (*P*_interaction_>0.13). It could be argued that the attenuated DNA methylation differences in conceptions towards the end of the famine period are related to changing external conditions. However, the daily rations remained very low and the average daily temperatures stayed above 0 °C from February onward (Fig. 4b), arguing for an intrinsic developmental explanation.

### Modestly sized famine associations extend into pathways

The six P-DMRs characterized in more detail were associated with relatively modest absolute differences between individuals from pre-April conceptions and sibling controls (4.2, 3.6, 3.2, −2.1, 4.1 and −6.1% for the P-DMRs mapping to *SMAD7*, *CDH23*, *INSR*, *RFTN1*, *CPT1A* and *KLF13*, respectively), reiterating the modest median within-pair difference found for the 181 P-DMRs as a whole. The functional impact of such potentially life-long differences remains to be established.

**Figure 5:**
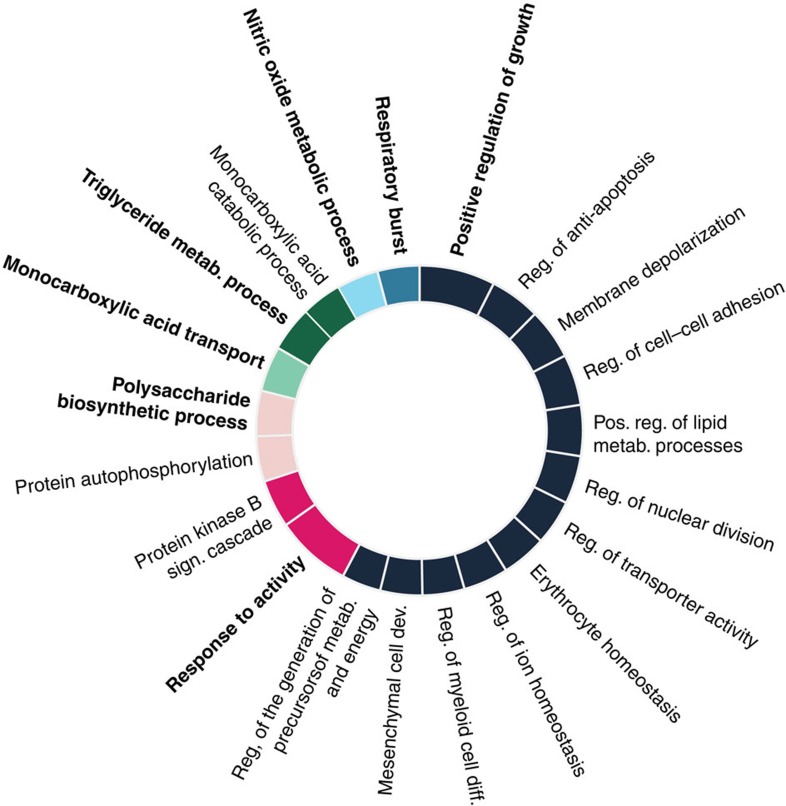
Revigo analysis of the significant pathways. A sunburst graph of the non-redundant clustered FDR significant GO terms associated with prenatal famine exposure. The size of the circular boxes are proportional to the level of statistical evidence. In bold are the dominant terms of the clusters, which are denoted in different colours.

Adaptive responses to cope with adverse intrauterine conditions were hypothesized to occur through multiple smaller changes across a gene network^33^, a hypothesis that recently gained empirical support^21^,^32^ and may explain how modest changes in DNA methylation may exert functional consequences. Therefore, we evaluated whether modest differential methylation at the six validated P-DMRs extended into biological pathways in which the nearest genes operate (Supplementary Fig. 1B.4). We revisited the genome-scale RRBS DNA methylation data in pre-April conceptions and their siblings and tested the association of DNA methylation with famine exposure along all MSigDB pathways and gene ontology biological processes (GO Fat) containing any of the six nearest genes. Before analysis, we removed all DNA methylation data mapping to these six genes (including the P-DMRs themselves) to achieve an unbiased analysis. DNA methylation signatures of 25 out of 101 pathways were associated with famine exposure (*P*_FDR_<0.05). The 21 significantly associated GO biological processes were clustered based on their relatedness and redundancy removed using REVIGO^34^. Visualization of these results showed that the largest cluster was formed from the GO biological process positive regulation of growth (Fig. 5). Multiple clusters contained pathways related to lipid and cholesterol metabolism (GO: positive regulation of lipid metabolic process , *P*_FDR_=0.028; lipid homeostasis, *P*_FDR_=0.042; triglyceride metabolic process, *P*_FDR_=0.049). To confirm the generality of these findings, we repeated the analysis across all 1637 MSigDB pathways and GO biological processes, and again observed an association between prenatal famine and the 25 pathways identified (*P*_FDR_<0.05).

### P-DMRs and phenotypic outcomes

The genes mapping to the six P-DMRs that were further characterized are involved in developmental processes including eye development (*CDH23* and *RFTN1*), forebrain formation (*SMAD7*), growth (*INSR*) and sustaining early pregnancy (*KLF13*). In addition, they have metabolic functions, including insulin signalling (*INSR*), pancreatic beta cell functioning (*SMAD7*), fatty acid oxidation (*CPTIA*) and cholesterol metabolism (*KLF13*). Thus, the function of these genes may potentially link development with phenotypic outcomes relevant for prenatal famine exposure^18^.

Individuals exposed early in gestation had higher birth weights than individuals born before and after the famine in the same institutions^6^, and replicated later-life outcomes include an unfavourable metabolic profile consisting of a higher BMI^35^,^36^, an altered glucose response^37^,^38^ and elevated LDL and total cholesterol levels^39^,^40^ for which data were available in our study. Associations between these five phenotypes and methylation at the six P-DMRs were compared in an exploratory analysis in the 60 sibling pairs measured with EpiTYPER (Supplementary Fig. 1B.5).

**Figure 6:**
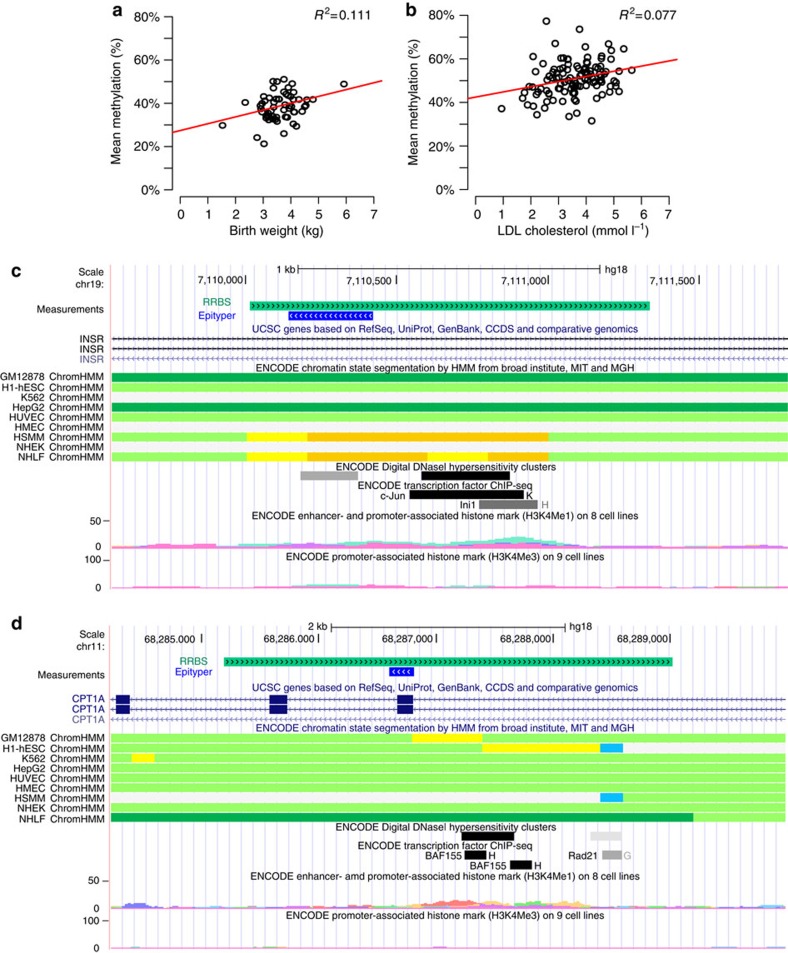
The INSR and CPT1A P-DMRs. (**a**) Scatterplot between birth weight (*x* axis) and the average DNA methylation of the *INSR* P-DMR (*y* axis) in the 60 prenatally exposed individuals. (**b**) Scatterplot between LDL (*x* axis) and the average DNA methylation of the *CPT1A* P-DMR (*y* axis) in all 120 siblings. (**c**) Genomic annotation of *INSR* DMR. The P-DMR overlaps an enhancer in the HSMM and NHLF cell lines and an DNaseI hypersensitivy cluster in over 30 cell lines. (**d**) Genomic annotation of *CPT1A* DMR. The P-DMR overlaps an enhancer in the blood derived GM12878 and embryonic stem cell line H1 and a DNaseI hypersensitive cluster in over 30 cell lines. Furthermore, the *BAF155* transcription factor binds in this region.

Birth weight could be investigated in exposed individuals only (*N*=60), since the same-sex sibling controls were not born at the institutions at which birth records could be retrieved. DNA methylation at the P-DMR at *INSR*, a gene intricately involved in prenatal growth, was positively correlated with birth weight (*R*^2^=0.111, *P*=9.0 × 10^−3^; Fig. 6a). The direction of the correlation was in line with the direction of the association of early gestational famine exposure with both birth weight and methylation at the P-DMR. The association remained statistically significant in a multivariate analysis that accounted for multiple testing (six P-DMRs and five phenotypic outcomes) and adjusted for the potential confounders gestational age, age at examination and adult characteristics such as SES, diet, BMI and smoking (*β*_exp-birth weight_=3.9%/1 kg, *P*_FDR_=0.033).

Of the later-life phenotypic outcomes tested (available in both exposed individuals and sibling controls, *N*=120), DNA methylation at the *CPT1A* P-DMR was positively correlated with LDL cholesterol levels (*R*^2^=0.077, *P*=3.5 × 10^−3^, Fig. 6b), which is in line with the direction of the association of early gestational famine with *CPT1A* methylation or LDL cholesterol independently. A similar association was observed for total cholesterol but this appeared to be mediated by LDL cholesterol. The association with *CPT1A* methylation and LDL cholesterol remained after correction for multiple testing and further adjustments for age, sex, BMI, SES, smoking and current diet (*β*_LDL_=2.4% per mmol l^−1^, *P*_FDR_=0.033). The effect size of the association was almost identical in the prenatally exposed individuals and their unexposed same-sex siblings (*β*_exp_=2.4% per mmol l^−1^; *β*_sibs_=2.3% per mmol l^−1^) and was not affected by exclusion of individuals using lipid lowering medication (*N*=−10; *β*_LDL_=2.5% per mmol l^−1^, *P*=2.5 × 10^−3^).

These phenotypic associations should be interpreted with caution and await replication. Furthermore, DNA methylation was measured in whole blood and although these P-DMRs did not overlap tDMRs^28^, we cannot confirm whether the DNA methylation patterns reflect those in more relevant tissues, for example, due to mitotic inheritance of differential methylation induced early in development, a phenomenon reported in model organisms^14^,^41^ and, recently, in humans^42^. The role of *INSR* on prenatal growth (and thus birth weight) is well established. How *CPT1A*, a rate-limiting enzyme in fatty acid oxidation, links to serum LDL cholesterol is less clear. Recently, two large population-based studies linked *CPT1A* gene body methylation (measured using the Illumina 450k array) with LDL cholesterol phenotypes^43^,^44^.

### Functional analysis of *CPT1A* and *INSR* P-DMRs

**Figure 7:**
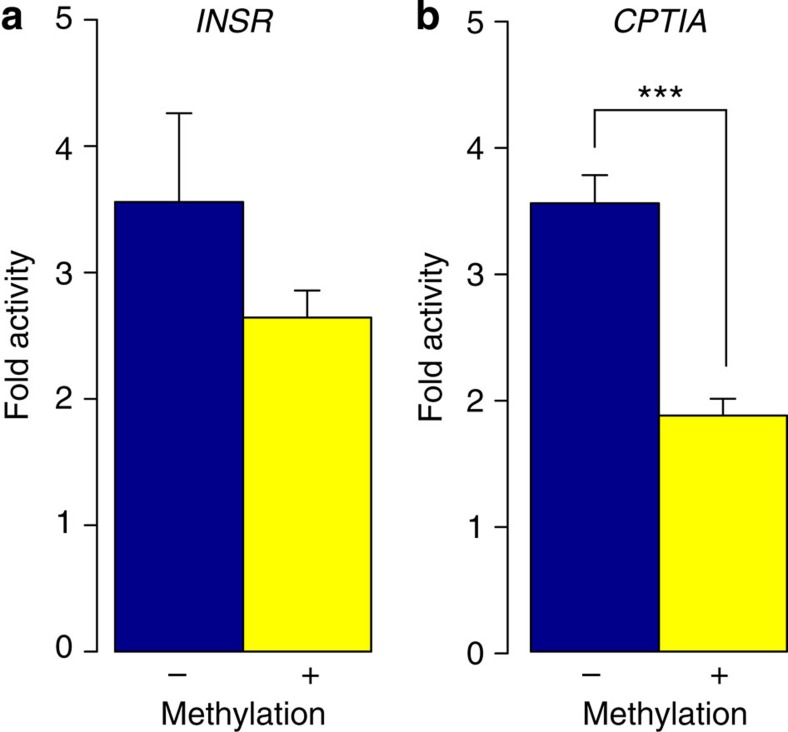
The difference in reporter gene expression for the INSR and CPTIA P-DMRs. The P-DMRs were inserted in front of an *EF1* promoter in the CpGL CpG-free vector. The *Luciferase* activity was normalized against *Renilla* activity. Next, the normalized fold change was calculated relative to activity of the CpGL-*EF1* vector, so the same vector but devoid of an enhancer. The blue bars denote the unmethylated vector set against the CpGL-*EF1* vector and the yellow bars the same vector, but now methylated, against the CpGL-*EF1* vector. Error bars denote the s.e. Each experiment was performed three times in duplicate. (**a**) The CpGL-*INSR*/*EF1* vector has a greater than threefold higher activity than the CpGL-*EF1* vector. DNA methylation of the *INSR* P-DMR does not result in a significant difference in activity as compared with the unmethylated CpGL-*INSR*/*EF1* vector. (**b**) The CpGL-*CPT1A*/*EF1* vector has a greater than threefold higher activity than the CpGL-*EF1* vector. DNA methylation of the *CPT1A* P-DMR results in a significantly lower activity of the vector as compared with the unmethylated CpGL-*CPT1A*/*EF1* vector.

Differential DNA methylation after prenatal famine exposure was identified at open chromatin regions and enhancers. All six characterized P-DMRs overlapped with one or multiple open chromatin regions and potential enhancers, as indicated by Broad HMM chromatin segmentation states (Fig. 6c,d, Supplementary Fig. 5A–D). We then focused on the intragenic *INSR* and *CPT1A* P-DMRs, on the grounds of their tentative associations with phenotypes. Both P-DMRs overlap a broad HMM enhancer state, which is not an uncommon observation, as most enhancers defined by chromatin segmentation states map to gene bodies (53%; Supplementary Fig. 7). To validate the predicted enhancer activity of the *INSR* and *CPT1A* P-DMRs *in vitro*, we cloned the regions in a CpG-free luciferase vector that harboured a CpG-free minimal promoter (Supplementary Fig. 1B.6)^45^, which was previously successfully used to evaluate enhancer activity and the influence of DNA methylation thereon^46^. We confirmed enhancer activity of the *INSR* P-DMR *in vitro*. Enhancer activity, however, was not modulated by DNA methylation (*P*=0.07, Fig. 7a) suggesting that differential methylation is not causally involved in regulating *INSR* P-DMR enhancer activity, but marks other epigenetic mechanisms or reflects a molecular remnant of differential gene expression during early development. The intragenic P-DMR at *CPT1A* overlaps a binding site of the *BAF155* transcription factor which is a repressor of self-renewal^47^ and is vital for early liver development^48^. The *CPT1A* P-DMR increased the expression of the reporter construct confirming its enhancer activity *in vitro* (Fig. 7b). DNA methylation of the P-DMR resulted in reduced reporter gene activity (*P*=3 × 10^−4^) indicating that DNA methylation can in principle affect enhancer activity although the (long-term) consequences of the subtle DNA methylation differences as we observed in this study remain to be established.

Together these data support a regulatory function of the P-DMRs, a subset of which may be directly regulated by DNA methylation.

## Discussion

Our genome-scale analysis identified key characteristics of genomic regions that are differentially methylated after exposure to malnutrition in early human development. We evaluated 28 potentially relevant genome annotations and observed that differential methylation occurred at regions with a regulatory role, including non-CGI promoters, open chromatin and enhancers that are active during the pre- and peri-implantation period^24^. The association with non-CGI promoters concurs with findings from a study on periconceptional folic acid supplementation and promoter methylation^49^. Within all the associated genomic annotations, we identified 181 individual P-DMRs. In line with the exposure period, the genes nearest to the P-DMRs were enriched for being expressed during early developmental phases. The majority of P-DMRs displayed intermediate DNA methylation levels. This is in line with findings from a large study on smoking-associated DNA methylation differences using the Illumina 450k array^50^. Intermediately methylated regions commonly show most interindividual variation and such regions are enriched for developmental processes and are hypothesized to be important in common diseases^51^. Also, a majority of P-DMRs occurred in gene bodies, reiterating findings from a mouse model of prenatal adversity^52^. This finding may reflect the large proportion of CpG dinucleotides in the human genome that map to gene bodies (51%) which in turn harbour a large number of regulatory sequences (53% of enhancers defined by ENCODE chromatin state segmentation are located within gene bodies).

Six P-DMRs mapping to *SMAD7*, *CDH23*, *INSR*, *RFTN1*, *CPT1A* and *KLF13* underwent technical and biological validation. Further analysis of the P-DMRs suggested the presence of a critical period or developmental phase after conception, possibly implantation^15^. Interestingly, differential methylation was not constrained to these individual P-DMRs but extended along the developmental and metabolic pathways in which their nearest genes are involved. In an exploratory analysis, we observed tentative associations of DNA methylation at the intragenic *INSR* and *CPT1A* P-DMRs with birth weight and lipid metabolism, respectively. Both P-DMRs were putative enhancers and we could confirm enhancer activity *in vitro*. *CPT1A* but not *INSR* enhancer activity was downregulated by DNA methylation.

While many studies have addressed the influence of prenatal adversity on DNA methylation in animal models, fewer have done so in human populations because of limitations associated with human research. The strengths of our study include that the setting of Dutch Hunger Winter can be regarded as a ‘natural experiment’ for the study of the prenatal environment in relation to metabolic disease in late middle age. The prenatal nutritional exposure is well defined and the prospective tracing of exposed individuals from birth records in famine-affected cities enables the relatively precise classification of gestational timing of the exposure^18^. Moreover, the use of unexposed same-sex siblings as control limits confounding from family environment and genetic factors. A major limitation of human studies is the common inability to study internal tissues that are most relevant for metabolic health. We performed various analyses to examine whether the differential methylation observed in DNA extracted from whole blood was nevertheless informative. For example, the 181 P-DMRs identified were not enriched for genes expressed in blood and a majority did not overlap with regions exhibiting tissue-specific DNA methylation (tDMRs), nor were the associations of the six validated P-DMRs confounded by blood cell counts. Interestingly, animal studies on prenatal malnutrition reported similar differential methylation in metabolic tissues at regions also observed in our study. We previously reported on differential methylation after prenatal famine exposure in whole blood at the *IGF2* (ref. 6) and *LEP*^7^ genes. Animal models of prenatal adversity likewise found differential methylation at these regions in metabolic tissues^53^ or both in metabolic tissues and in blood^41^. Vice versa, when we looked up the *HNF4A* promoter in our genome-scale data, which was previously found to be differentially methylated in pancreatic islets of rats after gestational protein restriction^54^, we observed a suggestive association with prenatal famine exposure in whole blood (*P*=0.05). However, none of the 181 P-DMRs overlapped with regions found differentially methylated in animal studies. This might be related to the differences in the type of exposure used in animal studies, coverage of the different methylation profiling methods, lack of conservation of the DNA sequence at P-DMRs across species, and the limited power of our study.

It has been postulated that differential methylation in whole blood may reflect that in other tissues through mitotic inheritance if induced early in development, a hypothesis that gained initial empirical support in model organisms^14^,^41^ and recently in humans^42^. The majority of the 181 P-DMRs did not overlap tDMRs, which may indicate that the DNA methylation patterns at these regions may be conserved across tissues, including those that play a role in metabolism such as in liver and adipose tissue. This may also have contributed to the observed differential methylation in pathways involved in development and metabolic regulation in our study on the basis of whole blood.

By design, our study could not provide a full catalogue of P-DMRs. First, whole-genome bisulfite sequencing is not yet feasible in an epidemiological setting and we used RRBS. Although delivering single-nucleotide resolution DNA methylation data on over a million CpG sites, it misses most of the in total 28M CpG sites in the human genome and, moreover, it is biased toward GC-rich regions. We excluded differences in GC content as source of the associations, even though the bias of RRBS remains apparent in the high coverage of GC-rich annotations as compared with CG-poor annotations. Nonetheless, also non-GC-rich annotations, like non-CGI promoters (28.9% covered), were covered by many individual regions so that such annotations could be reliably tested for an overall association with prenatal famine exposure. Second, the sample size in our genome-scale phase was limited to 48 individuals only which together with our chosen analysis strategy to reduce the multiple testing burden and maximize statistical power, will have caused us to miss many individual P-DMRs. A larger study with consequently higher statistical power combined with a more comprehensive technology to profile the methylome would have resulted in the identification of a substantial number of additional P-DMRs and a more comprehensive view of their overall genomic characteristics.

We are unable to assess whether initially larger DNA methylation differences waned over the six decades that passed between the exposure and DNA methylation measurements, nor can we exclude that the differential methylation observed has arisen over time. However, we did not notice an influence of age on DNA methylation patterns and the association of P-DMRs with prenatal famine was independent of current lifestyle. In view of their modest effect sizes, the importance of individual P-DMRs to phenotypic outcomes remains to be established. However, we observed that the modest effect sizes extended into the biological pathways their nearest gene was involved in. Understanding how multiple modest effects along pathways may together drive gene expression changes, as previously suggested for prenatal smoke exposure^21^, will be crucial to understand the role of epigenetic mechanisms in the developmental origins of disease.

The P-DMRs may reflect environmentally induced plasticity of the methylome, for example due to scarring or as an adaptive response to cope with intra-uterine adversity^55^. The observed pattern along the famine period may suggest that the induction of such differential methylation requires a prolonged exposure during early gestation to accumulate, or an exposure during specific developmental phases like those characterized by large-scale epigenetic programming (for example, around implantation^15^, 3-4 weeks post LMP in humans). However, the observed differential methylation in pathways related to energy production and growth may also be compatible with selection favouring the survival of embryos (or subsets of cells within an embryo) with a favourable epigenetic profile for growth under nutritional constraint. Nutritional constraint during pre-implantation development results in reduced cell numbers in pre- and post-implantation embryos^56^, presumably exacerbating the rates of early pregnancy loss that are already high under normal circumstances^57^.

Taken together, the DNA methylation signatures we describe are an important step towards cataloguing DNA methylation signatures of prenatal adversity that will be elemental in defining the contribution of epigenomic regulation in the aetiology of human disease.

## Methods

### Subjects

The Dutch Hunger Winter Families Study^18^ includes singletons born between February 1945 and March 1946 who had been exposed to famine *in utero*, births in 1943 without *in utero* exposure serving as prefamine time controls and births in 1947 without such exposure serving as post-famine time controls. Whenever possible, we recruited an unexposed same-sex sibling of each individual in this birth series to serve as a family control. Ethical approval for the study was obtained from the participating midwifery training schools in Amsterdam and Rotterdam and the University Medical Center in Leiden. All participants provided written informed consent and additional explicit approval for the current set of genome-scale measurements was given by the Leiden University Medical Center medical ethics committee.

Within the Hunger Winter Families Study, there are 313 same-sex sibling pairs who completed clinical examination. Sixty pairs included a sibling exposed to the famine around conception for up to 10 weeks into development (for example, ‘periconceptional’ exposure), as defined by a mothers’ estimated LMP between 28 November 1944 and 15 May 1945 (exposed: age 58.1, s.d. 0.35; unexposed same-sex siblings: age 57.1, s.d. 5.50). All 38 pairs of this group who had an age difference of less than 5 years at the time of examination were eligible for the genome-scale discovery phase. From these pairs we randomly selected 12 male and 12 female sibling pairs. Half of the female and half of the male pairs consisted of a sibling conceived and born after the famine as to prevent a consistent age difference between the prenatally exposed and controls and minimize a possible influence of early childhood famine exposure on the analyses (Supplementary Table 1).

### Phenotyping

Birth weights were only available for the individuals in the hospital series and were taken from the birth records from the three institutions at which these individuals were born, meaning that we do not have birth weight for the same-sex sibling controls. A telephone interview was performed with all participants and included questions on socio-demographic characteristics and medications for diabetes, cholesterol and blood pressure. The medical examinations were scheduled early in the morning after overnight fasting and included the measurement of height (to the nearest 1 mm by portable stadiometer (Seca, Hamburg, Germany)) and body weight (to the nearest 100 g by portable scale (Seca, Hamburg, Germany)). The BMI was calculated from these measurements. Participants had been asked to fast overnight before the clinic visit and underwent a standard glucose tolerance test with a 75-mg oral glucose challenge. Glucose was assayed in promptly separated serum by the hexokinase reaction on a Modular P800 (Roche, Boehringer Mannheim, Germany); the interassay coefficient of variation was 1.3–1.8%. The lowest detectable amount was 3 mU l^−1^. Serum total cholesterol, high-density lipoprotein cholesterol and triglycerides were collected from the baseline fasting blood draw and immediately measured by standard methods and LDL cholesterol was calculated for individuals with a triglyceride concentration lower than 400 mg dl^−1^ using the Friedewald formula.

### Sequencing and DNA methylation calling

Genomic DNA was extracted from whole blood using the salting-out method. We performed RRBS by following the published protocol^58^,^59^. Each of the 48 individual libraries was sequenced on a single Illumina GAIIx lane in the Broad Institute. An average of 25.6 million high-quality 36 bp single-end reads were obtained for each individual of which on average 74.1% (s.d. 10.4%) could be uniquely aligned to the bisulfite converted human genome (hg19) using custom scripts developed for RRBS data^59^.

Cytosines outside the CpG dinucleotide context were used to assess the bisulfite conversion rate. The average bisulfite conversion rate was 98.9% (s.d. 0.68%). The number of unmethylated and methylated cytosines per measured CpG dinucleotide was determined from the BAM alignment files using a custom python script^59^.

Mean methylation was assessed by calculating the average DNA methylation of all CpGs. The average methylation across all read was virtually identical in the exposed individuals and the sibling controls (42.55% and 42.64%, respectively; *P*=0.91). We also aligned all high-quality reads to the prototypic repeat sequences in the RepBase Update database and again determined the amount of methylated and total number of reads per consensus sequence CpG using custom software^19^. For each repeat type, the number of methylated and unmethylated reads was summed, repeats with a median coverage higher than five reads over all the measured sibling pairs were included in our analysis. The average methylation of repeat types may be viewed as a proxy of global methylation for which we previously tested five different assays based on repeats and found no differences in these same individuals^32^,^60^.

### Data grouping to genomic annotations

The location of CpG dinucleotides was first lifted to hg18 (NCBI36) since at the start of the study most annotations were available for hg18. All CpG dinucleotides were then mapped for an overlap with genomic features such as promoters and ‘bona fide’ CpG islands^22^, which are CpG islands (CGIs) with a ubiquitously open chromatin conformation. Most of the genomic feature annotations were taken from Gu *et al*.^13^ These annotations were supplemented with genome-wide annotations of enhancers^23^, CTCF-binding sites^61^, bivalent chromatin domains from human embryonic stem cells^62^ and human hematopoietic stem cells^63^, highly variable regions^64^, loci hypothesized to be sensitive to early nutrition (putative metastable epialleles)^65^ and enhancer regions shown to be associated with genes involved in pre- and peri-implantation development (dev. Enhancers Type I) or early differentiation stages (dev. Enhancers type II)^24^. Individual CpG sites were mapped to a specific genomic locus contained within the annotations of genomic features (for example, mapped to a particular promoter or other feature) when they had an overlap in terms of their genomic location. Each locus was subsequently mapped to the nearest entrez gene identifier within 100 kb.

### GlobalTest for genomic annotation and pathway analysis

We used the R package GlobalTest^66^ to test genomic annotation as a whole and to test groups of individual regions that mapped to gene ontology sets. This allowed us to comprehensively test both genomic annotations and pathways without having to rely on enrichment test, which may be biased for an enrichment method like RRBS. For this test, DNA methylation values within a given genomic region were transformed to yield normally distributed data that accounted for missing values, differences in the total coverage (thus accuracy of the measurement) and the number of CpG sites per locus. The transformation shrinks the fraction of methylated reads towards the average methylation fraction in the population of 48 subjects. This shrinkage is especially pronounced in subjects with few reads. The transformation was performed as follows:

*M*_*i,j*_: denotes the number of methylated reads for individual *i*, CpG dinucleotide *j* for a particular region.

*T*_*i,j*_: total number of reads for individual *i*, CpG dinucleotide *j* for a particular region.

*k*: the number of CpG sites for a given region (=*j*_max_)




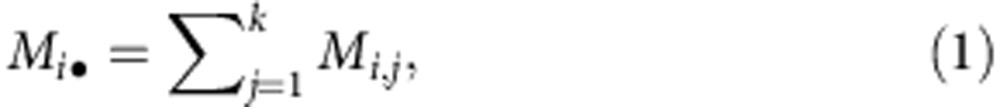




which denotes the sum of all methylated reads for individual *i* for a particular region.




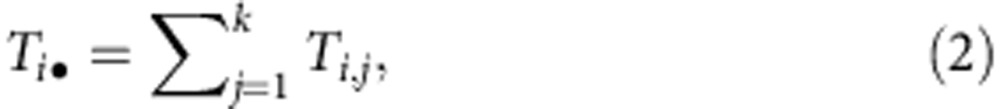




which denotes the sum of all reads for individual *i* for a particular region.




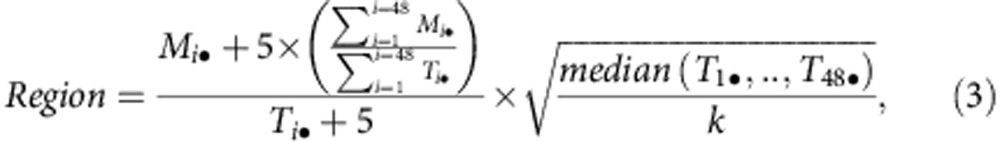




which denotes the final transformation of sequencing data of a genomic region.

As part of the validation of this approach, we tested the transformed values of the regions that were associated to prenatal famine exposure and a set of loci that were not or only nominally associated in our earlier candidate studies in this population^6^,^7^ by GlobalTest. The positive set was again associated with prenatal famine exposure (*P*=9 × 10^−3^) and the negative set was not (*P*=0.19).

### Enrichment tests on the 181 P-DMRs

All reported enrichment tests were performed by contrasting the 181 P-DMRs with the non-significant regions from the five annotations from which they were identified, as to prevent that the selection of the five significantly associated annotations affected the enrichment tests. All enrichments, outside those reported by using EPIGRAPH, were performed by Fisher’s exact tests using the R base Fisher.test() function.

### EpiTYPER data generation and pre-processing

Primers were designed using Methprimer. The resulting primer and amplicon locations were checked for SNPs with the latest version of dbSNP. The spectrum characteristics of all assays were checked with the R package RSeqMeth^67^. The sequences of the primers as well as the genomic locations they amplify are given in Supplementary Table 7. The regions measured are necessarily smaller than the regions identified by RRBS, the genomic overlap and the number of identical CpG dinucleotides measured is given in Supplementary Table 4.

Genomic DNA (1 μg) was bisulfite converted using the EZ 96-DNA methylation kit (Zymo Research) with overnight bisulfite incubation according to the supplier’s protocol. The 60 sibling pairs were randomly distributed over two 96-well plates with similar proportions of male and female pairs on each plate and in similar proportions for the pairs also measured with RRBS and those who were not. DNA methylation was quantitatively assessed for each locus using the mass spectrometry-based EpiTYPER assay (Agena Bioscience GmbH) in triplicate using the manufacturers’ protocol on one 384-well plate. PCR was performed with the following cycling protocol: 15 min at 95 °C, four rounds of 20 s at 95 °C, 30 s at 65 °C, 1 min at 72 °C; followed by 40 rounds, 20 s at 95 °C, 30 s at (see Supplementary Table 7)°C and 1 min at 72 °C; ending with 3 min at 72 °C. Processing of the EpiTYPER data was performed as reported previously^6^,^7^,^68^, namely fragments containing CpG dinucleotides (‘CpG units’) that have a mass within the mass range that do not overlap other CpG units were considered. Fragments were also discarded if dbSNP indicated the presence of a SNP in individuals of European decent with a minor allele frequency higher than 5%. Measurements for CpG dinucleotides containing fragments for which two out of three measurements were successful, the s.d. was smaller than 10% and for which the overall measurement success rate in the 60 pairs was higher than 75% were included in the analyses. Averages for these triplicate measurements were used for the analyses. For each measurement non-bisulfite converted genomic DNA and negative controls were incorporated to check for aspecific amplification and PCR artifacts. None were found. Bisulfite conversion was assessed using Sanger bisulfite sequencing and was ≥98% for both 96-well plates.

### Statistical tests for individual regions

We tested for within-pair differences in DNA methylation between exposed individuals and their same-sex siblings for individual loci by applying generalized linear mixed models on the sequencing data and linear mixed models for the EpiTYPER data. With these models, the correlation between adjacent CpG sites in an individual can be taken into account and all available raw but incomplete data can be used for modelling and control for possible confounders. The R programming environment was used for all analyses.

For the bisulfite sequencing data, we used logistic mixed models using the glmer() function from the lme4.0 package^69^ with a binomial distribution, weighting by the sequencing depth per individual observation. The dependent variable was the DNA methylation fraction. This is effectively the same as modelling each individual read as either 0 or 1 (unmethylated or methylated) as dependent variable without weighting for coverage depth, hence the application of a logistic model. Exposure status (exposed versus unexposed) and a unique identifier for each CpG dinucleotide were entered as fixed effects. To specify a within-sib-pair design, the (family) pair identifier was included as a random effect with intercept. To model the correlation in DNA methylation within an individual we make use of the fact that each family consists of an exposed individual and a same-sex sibling, therefore adding the exposure status to the model as a random slope, possibly correlated to the random intercept. This model is equivalent to the one in which the individual identifier was added as a random effect. The model allowed us to use the same model for both multiple CpG sites and single CpG sites.

For the continuous EpiTYPER data, we used a linear mixed model based on the lmer() function from the same lme4.0 package^69^, applying the same model used for the RRBS data, but now without the necessity to weigh for coverage and using a normal distribution. The models were fitted by REML or, when model fits were compared, by ML. The outcome of this model is exactly identical to a paired *t*-test if an individual CpG site is assessed, there is no missing data and no covariates are included.

We added bisulfite batch as fixed effect, since the 60 pairs were distributed across two 96-well plates for bisulfite treatment (keeping pairs on the same plate). Since the age difference is larger for some of the pairs in this set, age at blood draw was also entered as fixed effect. We are able to effectively correct for age because the siblings without prenatal famine exposure are equally distributed between being born before and after the war period. Additional corrections, like for possible confounders such as current diet or BMI, were performed by adding these respective variables as fixed effects to the model. For the current diet, we had data on the amount of kcal per day consumed and the percentage of fat, carbohydrates and protein in the diet. Interactions were tested by adding an interaction term as a fixed effect, always including the main terms. Models incorporating DNA methylation data from multiple loci were extended by removing exposure status as intercept and instead adding a random effect for individual with a nested random effect denoting the region. Normality of the EpiTYPER data was checked by histograms of the raw data and the lmer() model residuals. Model fits were diagnosed by plotting the residuals against the fitted values and comparing the variance of the residuals across all factorial covariates.

Multiple testing correction was performed according to the method developed by Bejamini and Hochberg, better known as ‘FDR’ (false discovery rate) correction using the R base ‘p.adjust()’ function. All *P* values reported are two sided. Reported confidence intervals are at 95%, without adjustment for multiple testing. The replication rate was calculated using the closed testing procedure based on the Simes inequality as described by Goeman and Solari^70^. The validation rate is an estimate of *π*_1_, the number of correctly rejected null hypotheses, and since its confidence interval is the most informative only, this was reported in the Results section. The *π*_1_ itself was 6/6.

### Reporter constructs

The *INSR* and *CPT1A* P-DMRs were inserted in the pCpGL-CMV/EF1 vector, which is devoid of CpG sites and was generously provided by the Rehli laboratory^45^. We replaced the CMV enhancer for the P-DMR, removing the CMV enhancer and linearizing the vector using PstI and SpeI (FastDigest, Fermentas). DNA sequences of the P-DMRs were attained from the UCSC Genome Browser (hg18) and primers were designed using Primer3 with slightly modified settings (primer size (18-20-22), primer Tm (59-60-61) and primer GC% (40-50-70). The resulting primers (Supplementary Table 8) were checked for self-complementary and dimer formation. We first performed a PCR on a region slightly larger than the P-DMR. After gel clean-up, this product was checked by Sanger sequencing. These products were subsequently used for a second PCR with primers to which a suitable restriction enzyme recognition site was added to insert the fragment in the linearized pCpGL-EF1 vector and an additional five basepairs (ATCAG) to provide sufficient physical space for the restriction enzyme to occupy the restriction site (Supplementary Table 8).

The PCRs were performed in 20 μl volumes with the following components and final concentrations: 12ul PCR buffer (Qiagen), 0.2 mM dNTPs Mix (Sequenom, Inc.), 2.5 ng μl^−1^ DNA (genomic (g)DNA, plasmid or PCR product), 0.25 pmol μl^−1^ forward and reverse primer (Metabion), 0.025 U μl^−1^ HotStarTaq (Qiagen). PCR reactions were performed in a DNA Engine Tetrad2 (Bio-Rad) according to the following programme: 15 min at 95 °C; four cycles of 30 s at 94 °C, 30 s annealing at 62 °C, 1 min at 72 °C; four cycles of 30 s at 94 °C, 30 s annealing at 58 °C, 1 min at 72 °C; 28 cycles of 30 s at 94 °C, 30 s annealing at 55 °C, 1 min at 72 °C and 8 min at 72 °C. After gel clean-up, the nested PCR products were cut using the restriction enzymes and ligated into the CpGL-EF1 vector using standard procedures and subsequently transfected to *E. Coli* GT115 (Invivogen) made competent by CaCl_2_. Clones were subsequently checked by clonal-PCR and Sanger sequencing using the primers denoted in Supplementary Table 10. A methylated CpGL-P-DMR/EF1 vector was created by treatment of the vector with M.SssI following the manufacturer’s protocol (NEB). Full methylation was confirmed by Sanger sequencing of bisulfite treated vector (Zymo Research) using primers denoted in Supplementary Table 9.

### Cell culture and (transient) transfections

HEK293 cells originally acquired form ATCC were cultured in Dulbecco’s modified Eagle’s medium (+4.5 g l^−1^ D-glucose, L-glutamate, pyruvate) (Gibco) with 10% fetal calf serum (Gibco) and 1% penicillin/streptomycin (Gibco) at 37 °C and 5% CO_2_. For transient transfection with the pCpGL vectors, HEK293 cells were used when the cell culture was 50% confluent. Cells were then seeded at a density of 100 K cells ml^−1^ in 12-well plates (1 ml per well) and left to grow for 6 h at 37 °C and 5% CO_2_ before transfection with Fugene HD (Promega). Transient transfection was performed by adding 50 μl containing 500 ng of methylated or unmethylated vector with a Fugene HD (μl) to DNA (μg) ratio of 3:1 (dissolved in Optimem (Gibco)) in accordance with the manufacturers’ suggested protocol, together with 5 μg of a Renilla vector (50:1). Cell lysates were created after 24 h by adding 250 μl of PLB (Promega) and 10 μl of cell lysate was used for the dual luciferase assay (Promega). Luciferase activity was normalized against Renilla activity and the activity of the CpGL-P-DMR/EF1 constructs was set out against the CpGL-EF1 constructs. The transfections were performed in duplicate in three consecutive experiments (in total six times). We performed linear regression with the fold activity values as dependent and the methylation status as fixed effect with a correction for the experiment.

## Additional information

**How to cite this article:** Tobi, E. W. *et al*. DNA methylation signatures link prenatal famine exposure to growth and metabolism. *Nat. Commun.* 5:5592 doi: 10.1038/ncomms6592 (2014).

**Accession codes:** Sequencing data (.BAM files) have been deposited in the European Genome-phenome Archive, which is hosted by the EBI, under the accession code EGAS0001000668. DNA methylation data have been deposited in the form of .BED files for each individual with the number of methylated reads and total reads per CpG sites in the Gene Expression Omnibus (GEO) under the accession code GSE54983.

## Supplementary Material

Supplementary Figures and Supplementary Tables Supplementary Figures 1-6 and Supplementary Tables 1-10

Supplementary Data 1 Overview of the 181 P-DMRs

## Figures and Tables

**Table 1:** Genomic annotation-centred analysis of differential methylation after early gestational famine exposure.

**Genomic annotations** *	**Covered with RRBS/total in genome (%)**	***P***_**nominal**_	***P***_**FDR**_
Non-CGI, ‘bona fide’ promoters†	2,024/7,014 (28.9%)	9.1 × 10^−4^	0.026
Enhancers‡	6,207/59,466 (10.4%)	1.9 × 10^−3^	0.026
DNaseI/FAIRE-seq regions§	79,728/590,252 (13.5%)	4.4 × 10^−3^	0.036
Middle exons	1,570/17,848 (8.8%)	5.8 × 10^−3^	0.036
Developmental enhancers type I||	922/5,118 (18.0%)	6.5 × 10^−3^	0.036
‘bona fide’ CGI shores¶	27,688/88,871 (31.2%)	0.012	0.053
Non-coding RNA#	59/718 (8.2%)	0.015	0.053
Conserved regions**	1,386/165,937 (0.8%)	0.016	0.053
CGI shores	67,811/319,509 (21.2%)	0.017	0.053
3′UTR	2,909/21,004 (13.8%)	0.035	0.085
Non genic CGI††	41,023/129,049 (31.8%)	0.036	0.085
‘Bonafide’ CGI border	22,777/88,074 (25.9%)	0.036	0.085
Developmental enhancer type II	320/2,287 (14.0%)	0.078	0.15
CGI	113,673/343,925 (33.1%)	0.078	0.15
Introns	61,816/201,640 (30.7%)	0.080	0.15
hESC bivalent chromatin domains	1,741/1,797 (96.9%)	0.16	0.28
Bonafide CGI	35,271/44,439 (79.4%)	0.20	0.32
Cell-type specific gene promoters	2,106/2,372 (88.8%)	0.21	0.32
First exons	13,507/51,497 (26.2%)	0.25	0.36
Promoters	16,904/23,689 (71.4%)	0.26	0.36
HSC bivalent chromatin domains	2,779/2,910 (95.5%)	0.28	0.36
Imprinted promoters	42/46 (91.3%)	0.29	0.36
‘Bona fide’ CGI promoter	14,880/16,674 (89.2%)	0.32	0.37
CTCF insulators from CD4+ cells	4,396/28,661 (15.3%)	0.32	0.37
Imprinted DMRs	6/14 (42.9%)	0.33	0.37
Putative metastable epialles	29/38 (76.3%)	0.43	0.47
Variably methylated regions	56/227 (24.7%)	0.55	0.57
Promoters cancer genes	795/888 (89.5%)	0.63	0.63

DMR, differentially methylated region; hESC, human embryonic stem cell; RRBS, reduced representation bisulfite sequencing; UTR, untranslated region.

The coverage of the genomic annotations is in line with the enrichment of RRBS for GC-rich regions, but also indicates a good coverage across annotations, including those relatively GC poor. *P* values were obtained with GlobalTest.

^*^More details on the genomic annotations can be found in the Methods.

^†^Promoters without CGIs but with a relatively open chromatin state^22^.

^‡^Enhancers characterized by H3K4me1, non-overlapping with promoters^23^.

^§^Regions with an open chromatin state as defined by DNaseI and FAIRE-seq signals (UCSC track ENCODE).

^||^Enhancers active during first stages of blastocyst development^24^.

^¶^Shores of the so-called bona fide CGI, CGI island with an ubiquitously open chromatin structure; oe>0.6, GC%>50% and length >700 bp^22^.

^#^Body of various type of non-coding RNAs.

^**^Conserved regions outside promoters, CGIs, exons and UTR.

^††^CGI>10 kb from gene.

**Table 2:** Technical validation of RRBS associations with EpiTYPER.

**Annotation** *	**Nearest gene (kb)**	**Discovery RRBS (** ***N*** **=48)**				**Validation EpiTYPER (*N*=120)**	
		**Methylated controls (%)**	**Within-pair difference (%)**	***P* value†**	***P*_fdr_**	**Within-pair difference (%)**	***P* value**
OC & Enh	*SMAD7* (+25)	21.3	4.2	1.0 × 10^−7^	1.1 × 10^−3^	3.2	2.5 × 10^−3^
OC & Enh	*CDH23* (0)	12.4	4.0	1.3 × 10^−7^	1.1 × 10^−3^	2.2	6.3 × 10^−3^
OC	*INSR* (0)	43.3	8.1	3.9 × 10^−6^	0.010	2.0	0.031
OC & Exon	*RFTN1* (0)	86.3	−2.3	3.2 × 10^−5^	0.030	−0.9	0.09
OC	*CPT1A* (0)	67.0	4.5	4.0 × 10^−5^	0.031	2.0	0.05
OC	*KLF13* (0)	67.1	−7.9	6.1 × 10^−5^	0.042	−3.1	0.014

Enh, enhancer; OC, open chromatin; RRBS, reduced representation bisulfite sequencing.

^*^Type of genomic annotations: OC, Enh and Exon.

^†^*P* value resulting from a linear mixed model.

**Table 3:** P-DMRs are specific for pre-April pregnancies.

**P-DMR**	**Discovery pre-April (*N*=36)** *		**Technical validation pre-April (*N*=36)** †		**Biological validation pre-April (*N*=36)** ‡		**April/May pregnancies (*N*=48)** §	
	**Difference**	***P* value**	**Difference**	***P* value**	**Difference**	***P* value**	**Difference**	***P* value**
* SMAD7 *	4.3	2.2 × l0^−7^	3.8	0.010	4.4	0.027	l.6	0.39
* CDH23 *	4.2	4.5 × l0^−6^	3.6	l.4 × l0^−3^	3.6	0.034	0.l	0.9l
* INSR *	9.0	7.4 × 10^−7^	2.5	0.l6	3.8	0.0l6	0.5	0.77
* RFTN1 *	−3.7	l.l × lO^−7^	−2.3	3.5 × l0^−3^	−l.9	0.l3	0.8	0.2l
* CPT1A *	4.9	1.1 × 10^−5^	4.4	0.04l	3.8	0.02l	−l.l	0.49
* KLF13 *	−8.6	l.6 × l0^−5^	−5.5	7.8 × l0^−3^	−6.0	7.5 × l0^−3^	l.0	0.57

P-DMR, prenatal malnutrition-associated differentially methylated region.

^*^Outcome of the generalized mixed effects model for a famine association for the pairs with one sibling conceived from November 1944 to March 1945 (18 pairs, 36 individuals) in the RRBS data.

^†^Outcome of the linear mixed effects model for a famine association for the EpiTYPER measurements on the same pairs as measured by RRBS and with a sibling conceived between November 1944 and March 1945.

^‡^Outcome of the linear mixed effects model for a famine association for the EpiTYPER measurements for pairs not measured by RRBS but with one sibling conceived between November 1944 and March 1945 (also 18 pairs, 36 individuals).

^§^Outcome of the linear mixed effects model for a famine association for the EpiTYPER measurements for all pairs with one sibling conceived in April or May 1945 (24 pairs, 48 individuals, of which six pairs were also included in the RRBS discovery measurement).
