# Supplementary material for: DNA methylation signatures link prenatal famine exposure to growth and metabolism
Source: Nat Commun. 2014 Nov 26;5:5592. doi: 10.1038/ncomms6592 (PMC4246417; doi:10.1038/ncomms6592)
Supplement: Supplementary Figures and Supplementary Tables — Supplementary Figures 1-6 and Supplementary Tables 1-10 [file ncomms6592-s1.pdf]

Supplementary Information

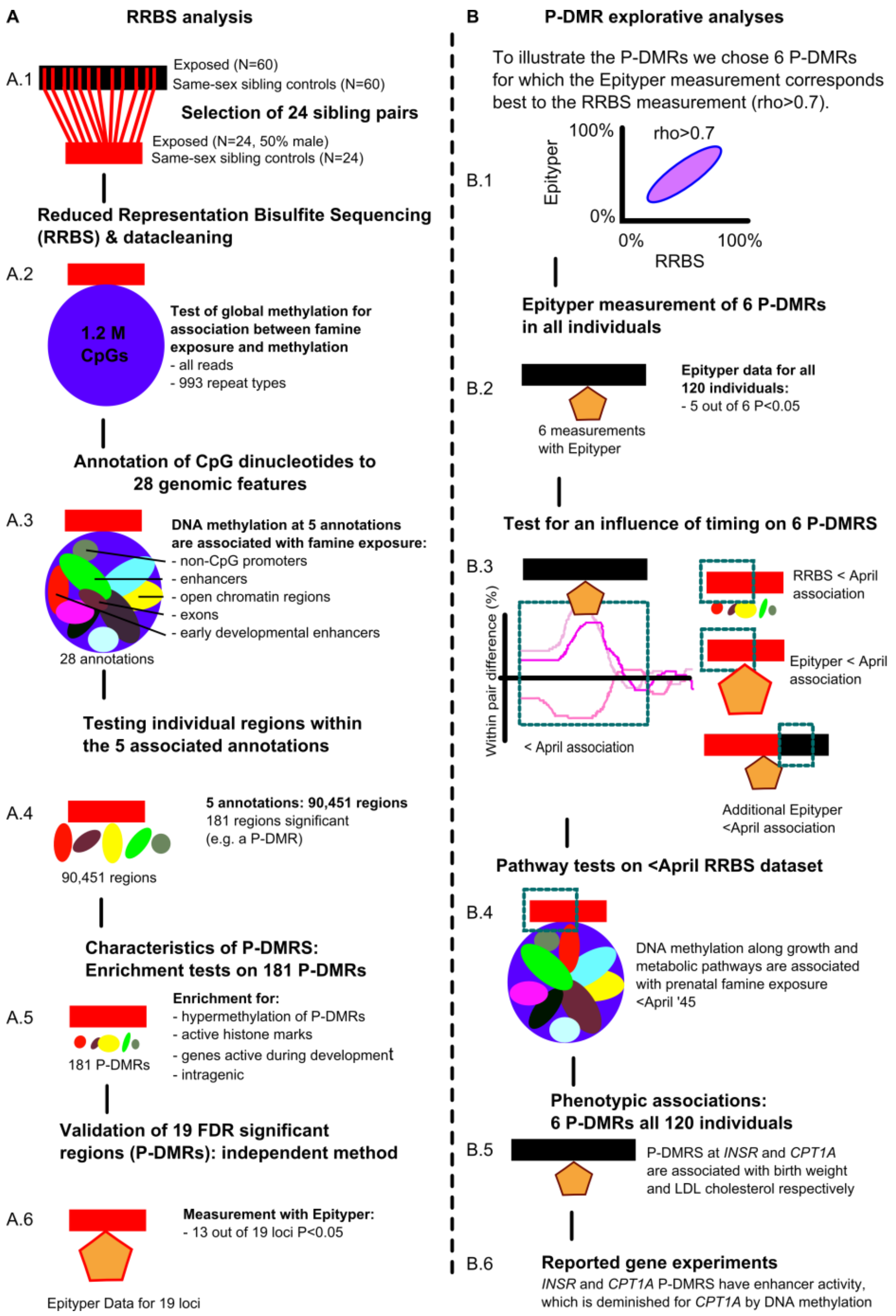

Supplementary Figure 1. Flow chart.

Overview of the experimental workflow. Starting with the selection of individuals for RRBS, data cleaning and genome-scale analyses (A) and region specific analyses (B).

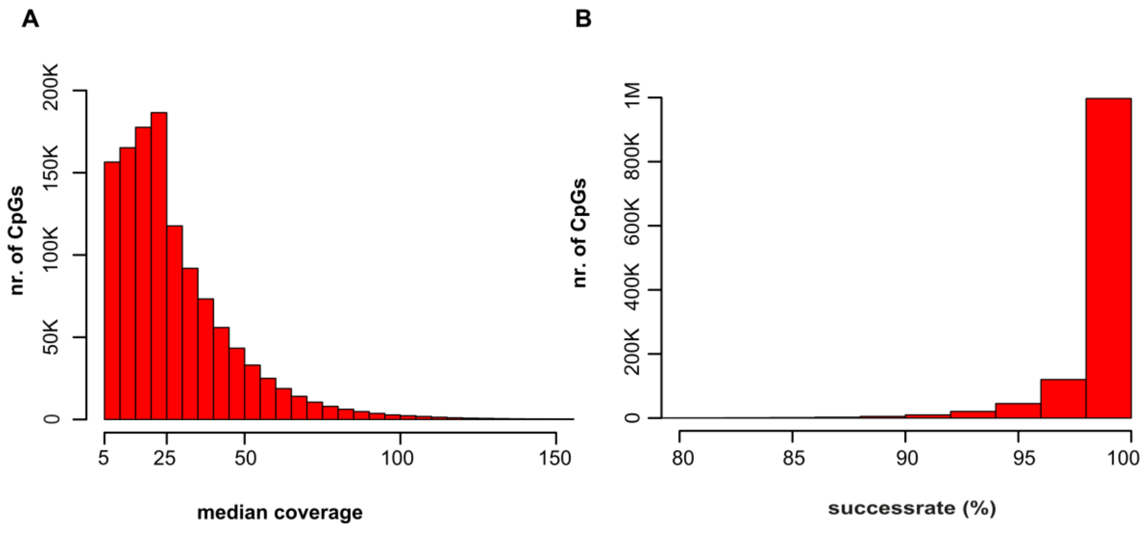

Supplementary Figure 2. Histograms of the median coverage and success rate per CpG dinucleotide

A. Histogram of the median coverage (e.g. sequencing depth) over the 48 individuals of the CpG dinucleotides included in the analyses (N=1.206.149).
B. Histogram of the success rate for each CpG CpG dinucleotides included in the analyses for the 48 individuals.

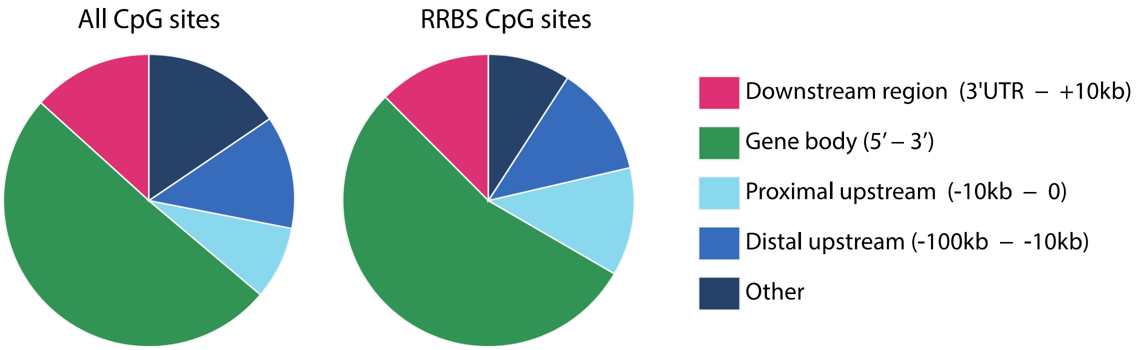

Supplementary Figure 3. Distribution of all (28.2M) and RRBS-interrogated (1.2M) CpG dinucleotides across the human genome relative to genes

Pie charts depicting the percentage of the CpG sites contained within different genomic features, namely regions downstream of genes (Downstream region), the genes themselves (Gene body), proximal promoters (Proximal upstream) and more distal upstream regions (Distal upstream). All CpGs located more than 100kb upstream and more than 10kb downstream of an Entrez Gene ID are designated als ‘other’. The left pie chart depicts the distribution of all ~28M CpG sites in the human genome, while the pie chart on the right the 1.2M CpG dinucleotides measured with RRBS after data cleaning. The distribution is remarkably similar, only a slight depletion of ‘other’ is seen.

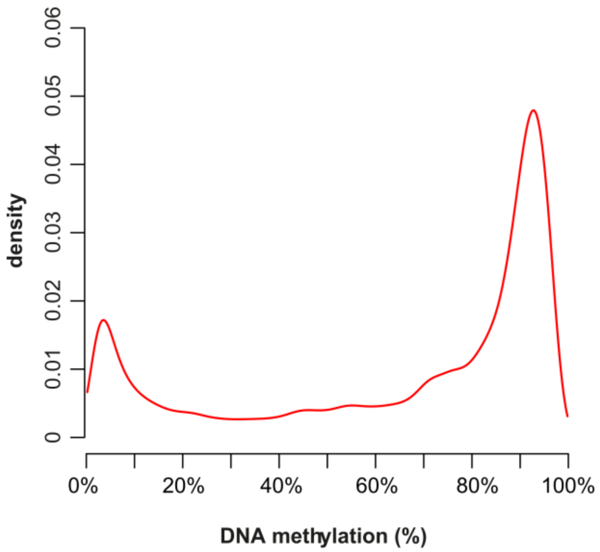

Supplementary Figure 4. Density plot of the average methylation of the CpG dinucleotides

Density plot of mean methylation of all 1.2 M CpG dinucleotides after data filtering. All CpG dinucleotides with a median methylation of 0% or 100% were removed; however, the classical bimodal distribution of DNA methylation is still apparent.

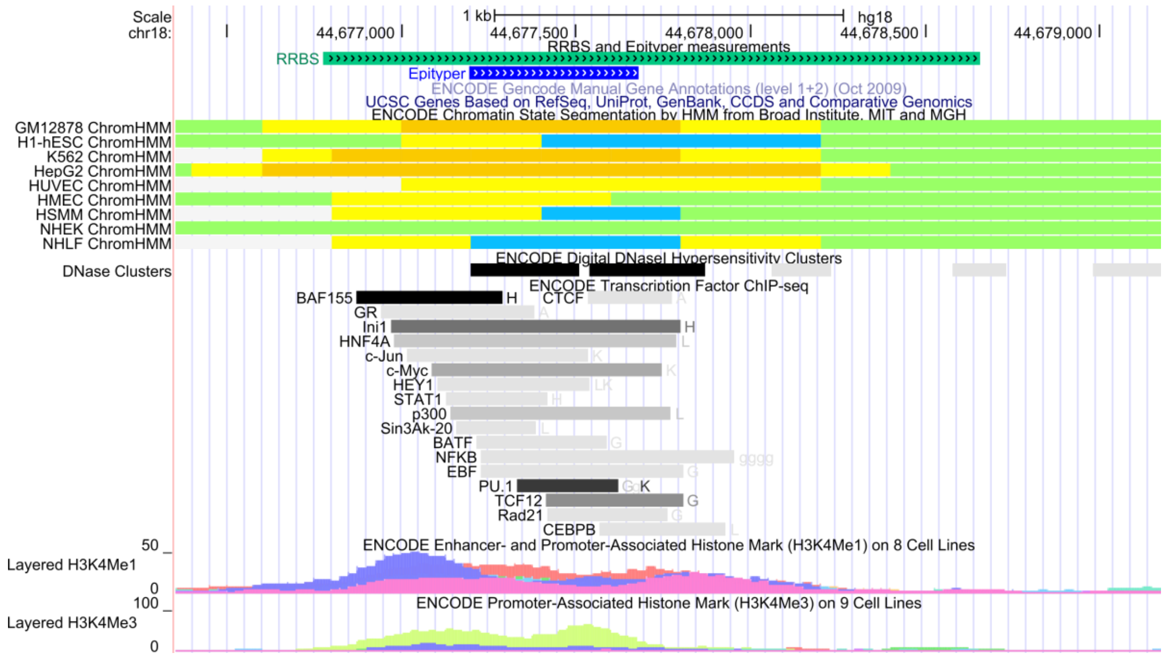

Supplementary Figure 5A. The SMAD7 P-DMR

The RRBS and Epityper measurements are located in the enhancer (orange/yellow) and isolator (blue) histone state at a strong DNaseI hypersensitivity locus in over 30 cell lines and overlapping various transcription factor binding sites, including the DNA methylation sensitive PU.1, HNF4A, p300, CTCF, CEBPB and NFKB factors.

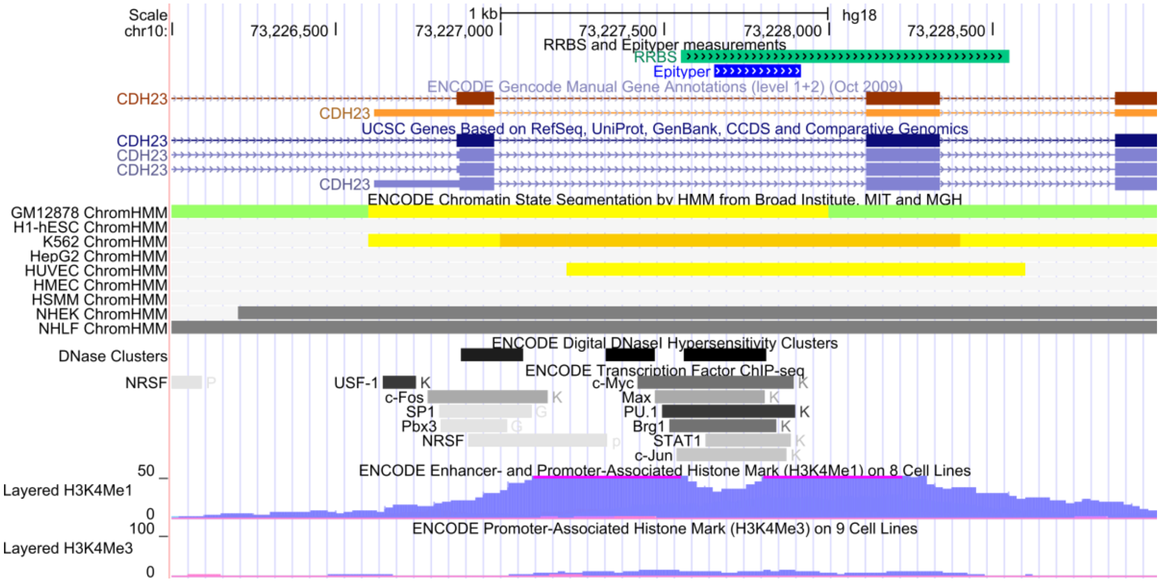

Supplementary Figure 5B. The CDH23 P-DMR

The RRBS and Epityper measurements overlap part of an enhancer (orange/yellow) histone state in multiple cell lines, including one derived from blood (GM12787) and liver (K562). The locus overlaps a hypersensitivity locus in over 30 cell lines and overlaps with various methylation sensitive transcription factors, including PU.1.

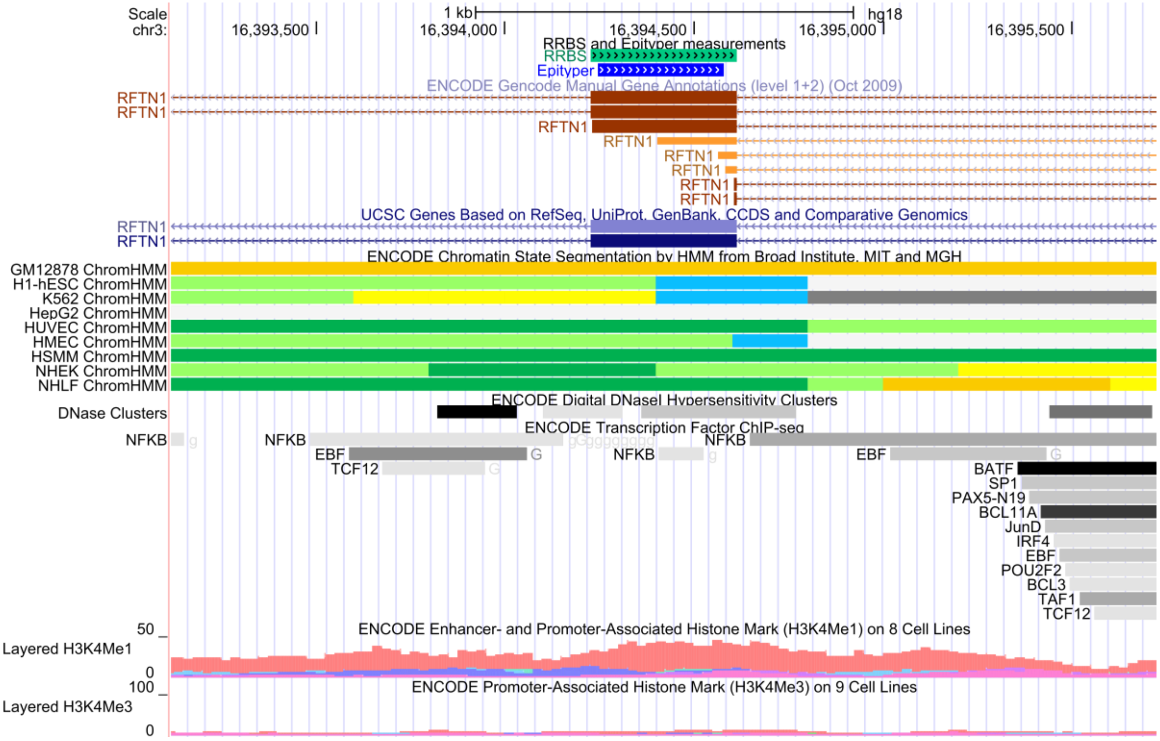

Supplementary Figure 5C. The RFTN1 P-DMR

The P-DMR overlaps an exon or the 5’-UTR from various *RFTN1* isoforms at a site characterized as a strong enhancer (orange) or isolator (blue) histone state in various cell lines.

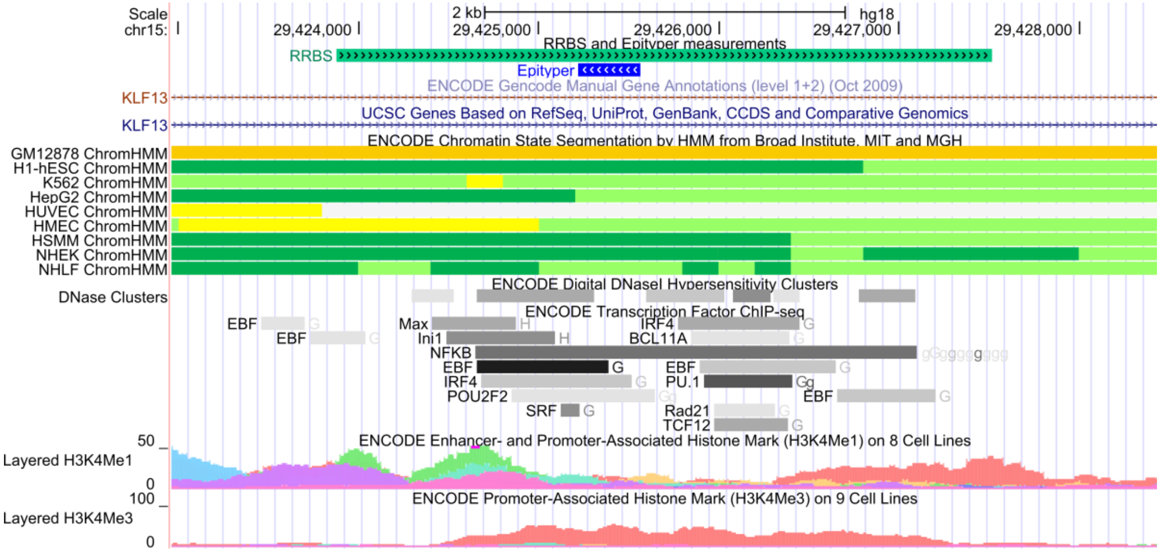

Supplementary Figure 5D. The KLF13 P-DMR

The P-DMR overlaps an intron of *KLF13* at a site defined as a strong enhancer (orange) histone state and overlaps DNaseI hypersensitive sites in various cell lines and overlaps methylation sensitive transcription factor binding sites for POU2F2 and PU.1.

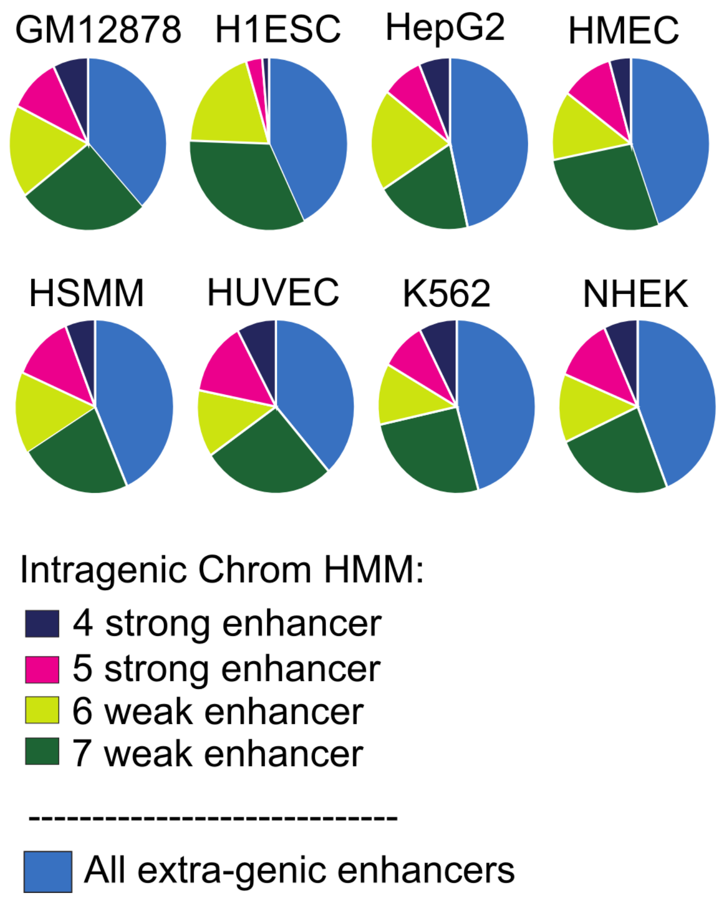

Supplementary Figure 6. Proportion of intragenic enhancers in core ENCODE cell lines

Each pie-chart represents one ENCODE cell line. Different enhancer states as defined by chromHMM are marked by different colors. All enhancers outside gene bodies have been colored light blue. Clear is that intragenic enhancers are slightly more common than extra genic enhancers in all these cell lines.

Supplementary Table 1. Population and sequencing characteristics

Variable	Quantity
Individuals sequenced	48
Same-sex sibling controls	24
Age (SD)	58.3y (2.1)
Percentage of males	50%
Number of pre-war born sibling	12
Male pre-war born siblings	6
Median quality score reads (SD)	35.3 (2.0)
High quality reads million (SD)	25.6 (7.3)
Reads mapped uniquely (SD)	74.1%(10.7)
Bisulfite conversion (SD)	98.9%(0.7)

Supplementary Table 2. Data filtering steps

Filtering steps	Total CpGs	CpGs matching
Total unique CpGs	3.174.757	
Random chromosome		3.195
Median Coverage <=5		1.296.450
Median Coverage >200		2.935
Median methylation = 0%		1.400.875
Median methylation = 100%		252.439
Total included unique CpGs*	1.206.149	

*Considerable numbers of CpG dinucleotides match multiple filtering criteria, resulting in a final number of included CpG dinucleotides higher than the subtraction of the 'CpGs matching' column form the total unique CpGs.

Supplementary Table 3. Epityper associations with prenatal famine

Annotation		Chosen by:	RRBS (N=48)				RRBS-Epityper	Epityper (N=48)	
Feature type[1]	Nearest Gene	criterion	Mean meth. (%)	Within pair diff. (%)	P[2]	P_{FDR}	correlation[3]	Within pair diff.(%)	P[4]
1	*DHRS4L2*	Lowest p-val	3.7	1.8	$3.0x10^{-9}$	$2.5x10^{-4}$	0.27	0.4	0.71
2	*EHD1*	2nd	45.7	-5.2	$5.7x10^{-9}$	$2.5x10^{-4}$	0.50	-4.0	$2.2x10^{-4}$
2	*DAPK2*	3rd	67.1	4.5	$1.1x10^{-8}$	$2.6x10^{-4}$	0.57	1.9	$6.4x10^{-3}$
2 & 3	*LOC554202*	4th	24.2	6.5	$1.7x10^{-8}$	$2.6x10^{-4}$	0.52	3.5	$3.2x10^{-3}$
2	*LSM14B*	5th	52.0	3.5	$1.7x10^{-8}$	$2.6x10^{-4}$	0.17	1.1	0.24
2	*ASS1*	6th	68.2	-4.9	$4.8x10^{-8}$	$6.2x10^{-4}$	0.22	-0.5	0.42
2	*SMAD7*	7th	21.3	4.2	$1.0x10^{-7}$	$1.1x10^{-3}$	0.78	4.7	$3.0x10^{-4}$
2 & 3	*CDH23*	8th	12.4	4	$1.3x10^{-7}$	$1.1x10^{-3}$	0.73	3.9	$8.1x10^{-5}$
2	*MIR4315-1*	9th	30.2	8.9	$2.1x10^{-7}$	$1.7x10^{-3}$	0.47	2.0	0.077
2	*ZIC1*	11th	5.9	1.4	$3.2x10^{-7}$	$2.1x10^{-3}$	0.49	0.6	0.011
2	*INSR*	Chosen	43.3	8.1	$3.9x10^{-6}$	0.01	0.72	3.9	0.018
2	*HOXD3*	Chosen	13.5	3.2	$4.3x10^{-6}$	0.01	0.26	1.2	0.066
2	*RPTOR*	Chosen	83.1	1.7	$9.1x10^{-6}$	0.02	0.63	8.6	$2.4x10^{-4}$
2 & 4	*RFTN1*	Chosen	86.3	-2.3	$3.2x10^{-5}$	0.03	0.71	-1.9	$2.4x10^{-3}$
2	*STX1A*	Chosen	29.1	3.7	$3.2x10^{-5}$	0.03	-0.17	1.1	0.17
2	*CPT1A*	Chosen	67.0	4.5	$4.0x10^{-5}$	0.03	0.70	4.0	0.015
2	*SCARB1*	Chosen	74.7	-10.5	$5.3x10^{-5}$	0.04	0.55	-4.9	$1.7x10^{-4}$
2	*KLF13*	Chosen	67.1	-7.9	$6.1x10^{-5}$	0.04	0.79	-5.7	$7.9x10^{-4}$
2	*KLF6*	Chosen	44.9	-6.2	$7.9x10^{-5}$	0.046	0.52	-2.6	$2.6x10^{-4}$

1. Type of genomic feature, 1 = non-CGI 'bonafide' promoter, 2 = Open chromatin, 3= enhancer, 4 = exon.
2. Two-sided P value resulting from a generalized linear mixed model where the analysis was weighted for sequencing depth.
3. The Pearson correlation between the average methylation of the RRBS region and the average of the CpG dinucleotides measured in the smaller region measured by Epityper.
4. The P value coming from a linear mixed model using the DNA methylation data generated with Epityper.

Supplementary Table 4. EpiTYPER primers

NR[1]	Locus		Sequence
0	HUGO	Strand	FORWARD[3] (5'-3')
	coordinates(hg18)	T (°C)[2]	REVERSE[4] (5'-3')
1	DHRS4L2	-	GAGGATAGGGGTATTGGAGGTAAAG
	chr14:23,527,972-23,528,213	53	AAACCCAAACTTACTAATCTAATCCATA
2	EDH1	+	TTTGTTGTGAGGGAAATATAGTGATTG
	chr11:64,389,159-64,389,510	48	CCCTACCTTAATAAATACCAAAACCTAAA
3	DAPK2	-	TGATGATTTAATTTTGTGGGTTTGTGT
	chr15:62,062,839-62,063,043	53	AAATCCTAAAAACCCCACTCACAACT
4	LOC554202	+	AATTATTGGAGTGTAAAATTTTTTT
	chr9:21,595,548-21,595,759	48	CTATTTCCTAATAACCCATATTTAC
5	LSM14B	+	TTGTTTAGGAGGGTTTATTTTATGGTT
	chr20:60,141,366-60,141,715	53	ATAACAAACACTAACTCCCAAACTTCTAAC
6	ASS1	-	TGTTTTAGGGTGGGTATAGTTTAGGT
	chr9:132,344,966-132,345,523	46	ACCAAACCTCCTTAAAACTCTTCATA
7	SMAD7	+	TTGGGTTATATTTTATGTGTGGTGT
	chr18:44,677,194-44,677,679	53	CAACAACTAACTCTTTCCTACATCTAACT
8	CDH23	+	ATAGGGGAAGTTAGGTTTGGTAGAT
	chr10:73,227,653-73,227,914	53	ACTAAATAACCCTAATAAAACCCTC

9	miR4315-1	+	TTTTTTTTGTTTTTGATAGGGTTATG
	chr17:55,579,661-55,579,969	53	CCCAATATTCTAAATTCAAATCTTTACTCT
10	HIF1AN	-	AAAAGTGTTGATAGGGTTAGGAGAG
	chr10:102,319,419-102,319,893	Not working	TTTTATATATAACTTAATATAACAACATCA
11	ZIC1	+	TTTGGGTTTTTTGTTTTTAAGAGGT
	chr3:148,612,363-148,612,675	53	ACTTCCACCTAACTCCTAATTTCTAATTTT
C1	INSR	-	TTTTTAGGAGGTTTTTAGAGTTTTTAGATT
	chr19:7,110,140-7,110,418	44	CTAACCTCAAAATAATCCACCCCAC
C2	KLF13	-	AGGTAGGTATTTGTATAGAGGGGTTTA
	chr15:29,425,223-29,425,563	53	AAACTAACCACACCAAAACTTAATATACTT
C3	STX1A	+	GGTGAGGGGTTATAGATTAGGAGGT
	chr7:72,759,326-72,759,710	53	AAACAAACTAACCAAACAAACAAAACT
C4	RPTOR	-	GTTGGATGAGTAGGTTTTGGATGG
	chr17:76,479,050-76,479,293	46	CAATTCATAAAACAAAAAAAATTTAAAATA
C5	CPT1A	-	TTTAGGATATGGGTAAGTTTTGTTTTATAT
	chr11:68,286,598-68,286,810	48	AAATAATAACCTCCAAAAACCTTTTAAAAA
C6	RFTN1	+	TATGTATTTTTAAGGGGTTGTTTTT
	chr3:16,394,247-16,394,578	48	TAATATTATACCTCAATACCATTCCTCTAT
C7	HOXD3	+	TTTGGTGGTTTAATTTTGGTTAATT
	chr2:176,735,594-	48	ATAAAAACATCCCCTCAAAAAAAA

	176,735,816		
C8	KLF6	-	AATAGTTTGAATTTAGATGTTAGTAG
	chr10:3,813,737-3,814,216	46	CCAAAACTAATACAATACAATAAC
C9	SCARB1	+	GGTGGTTAGGGTTAGTAAGAGAAGTA
	chr12:123,789,477-123,789,580	53	CCTATAACTCAAACTTCAAAAAAAAC

1. Primer pair number corresponding to the lowest p-value in RRBS (nr1-11; no reliable PCR was possible for nr10, chr10:102319110-102321355 [*HIF1AN*]), several regions were chosen (C1-C9).
2. Sequence of the forward PCR primer: for epiTYPER a tag with the following sequence is added 5’: 5’-AGGAAGAGAG-sequence.
3. The annealing temperature in the PCR program: 15 min at 95°c, 15 minutes at 95°C, 4 rounds of 20 seconds at 95°C, 30 seconds at 65°C, 1 minute at 72°C; followed by 40 rounds, 20 seconds at 95°C, 30 seconds at ***Ann.T*** and 1 minute at 72°C; ending with 3 minute s at 72°C.
4. The sequence of the reverse primer, for epiTYPER a tag with the following sequence is added 5’: 5’-CAGTAATACGACTCACTATAGGGAGAAGGCT-sequence

Supplementary Table 5. Number of CpG sites measured, and overlap between RRBS and Epityper measurement

Nearest Gene	location RRBS[1]	Location Epityper[2]	Nr CpGs RRBS	Nr CpGs Epityper	Nr CpGs overlapping between assays
DHRS4L2	chr14:23,526,866-23,528,866	chr14:23,527,972-23,528,213	5	8	5
EHD1	chr11:64,374,355-64,390,875	chr11:64,389,159-64,389,510	26	9	8
DAPK2	chr15:62,060,557-62,063,275	chr15:62,062,839-62,063,043	7	12	2
LOC554202	chr9:21,594,650-21,595,650	chr9:21,595,548-21,595,759	3	3	2
LSM14B	chr20:60,141,215-60,146,975	chr20:60,141,366-60,141,715	7	6	3
ASS1	chr9:132,344,794-132,346,579	chr9:132,344,966-132,345,523	12	14	3
SMAD7	chr18:44,676,775-44,678,655	chr18:44,677,194-44,677,679	7	7	4
CDH23	chr10:73,227,550-73,228,550	chr10:73,227,653-73,227,914	3	4	3
miR4315	chr17:55,579,535-55,580,790	chr17:55,579,661-55,579,969	3	6	3
ZIC1	chr3:148,611,075-148,612,495	chr3:148,612,363-148,612,675	21	14	5
INSR	chr19:7,110,011-7,111,334	chr19:7,110,140-7,110,418	2	3	2
KLF13	chr15:29,423,875-29,427,520	chr15:29,425,223-29,425,563	8	6	6

STX1A	chr7:72,758,075-72,760,255	chr7:72,759,326-72,759,710	7	8	4
RPTOR	chr17:76,471,363-76,483,344	chr17:76,479,050-76,479,293	45	6	3
CPT1A	chr11:68,285,186-68,289,024	chr11:68,286,598-68,286,810	15	8	7
RFTN1	chr3: 16,289,024-16,394,613	chr3:16,394,247-16,394,578	10	11	5
HOXD3	chr2:176,734,540-176,735,745	chr2:176,735,594-176,735,816	7	6	2
KLF6	chr10:3,810,815-3,816,315	chr10:3,813,737-3,814,216	20	18	10
SCARB1	chr12:123,788,495-123,792,067	chr12:123,789,477-123,789,580	6	6	4

1. Genomic location of the genomic feature, as used for the original analysis of the RRBS data.
2. Genomic location of the Epityper PCR amplicon.

Supplementary Table 6. Validation of the within pair differences in all 60 sibships with EpiTYPER.

	Exposed		Unexposed				
CDH23[1]	Meth. (%)	SD(%)	Meth. (%)	SD(%)	Success rate (%)[2]	Diff.[3]	P[4]
CpG_1	24.8	5.6	22.4	6.1	100	2.3	0.0079
CpG_2	22.6	6.2	20.5	6.1	100	2.1	0.019
CpG_3	19.8	5.1	17.5	5.2	100	2.2	0.0048
CpG_4	22.5	6.1	20.1	6.3	100	2.3	0.0078
SMAD7							
CpG_1	23.7	6.9	20.6	7.1	95	3.0	0.0052
CpG_2	22.6	6.4	20.3	5.9	96.7	2.3	0.014
CpG_3	18.9	7.4	15.6	7.3	96.7	3.1	0.0077
CpG_4	14	5.8	10.6	5.9	94.2	3.3	0.0034
CpG_5.6	20.7	5.7	18.3	5.9	96.7	2.4	0.013
CpG_7	23	7.1	20.5	7.8	91.7	2.6	0.046
INSR							
CpG_2	33.5	5.6	30.6	5.8	95.8	2.9	0.0031
CpG_3	40	7.2	38	6	86.7	1.7	0.13
CpG_5	43.4	7.3	41.7	6.5	91.7	1.7	0.15
KLF13							
CpG_2	75.8	9.5	79.4	11.3	77.5	-3.7	0.07
CpG_4	65.4	7.8	68.4	8.6	99.2	-3.0	0.019
CpG_5	67.2	7.1	70.2	8.5	100	-2.9	0.016
CpG_6	64.2	7	67.4	9	100	-3.2	0.015
CpG_7	58.2	6.7	61.1	8.2	98.3	-2.8	0.020
CpG_9	62.3	7	65.5	8.5	98.3	-3.1	0.013
RFTN1							
CpG_1	89.6	3	90.3	3.1	100	-0.7	0.13
CpG_2	85	3.6	85.6	3.5	100	-0.7	0.19
CpG_3	81.5	3.7	82.8	3.5	100	-1.2	0.046
CpG_4	90.5	4.7	91.6	3.3	86.7	-0.9	0.23
CpG_5	82.3	2.8	83.2	2.7	100	-0.9	0.054

CpG_6.7	92.9	2.8	93.5	2.6	100	-0.6	0.21
CpG_8.9	84.1	3.8	85.2	5.1	100	-0.9	0.21
CpG_10	81.6	4.8	82.8	4.9	100	-1.2	0.12
CpG_12	81.8	4	83	4.2	100	-1.2	0.07
CPT1A							
CpG_2.3	41.3	10.9	39.1	11.2	99.2	2.4	0.09
CpG_5.6	58.1	8.9	56.3	8.9	99.2	1.9	0.11
CpG_8.9	76.5	3.8	75.4	4	99.2	1.1	0.05
CpG_10	49.1	8.8	46.9	8.7	99.2	2.4	0.032
CpG_12	34	8	31.7	8.8	99.2	2.4	0.037

1. The locus and individual CpG sites measured with Epityper after data filtering. The CpG dinucleotides are measured from the forward primer onward.
2. The success rate of the measurement in the 120 individuals
3. The average within pair differences in the 60 sib ships
   The two-sided P-value resulting from a linear mixed model

Supplementary Table 7. Neutrophil variation and DNA methylation at the P-DMRs

P-DMR	Beta[1] (%methylation/ %neutrophils)	P[2]	SD change required to explain famine association[3]
SMAD7	-0.19	0.17	-5.1
CDH23	-0.35	$3.1x10^{-3}$	-1.9
INSR	0.02	0.91	31.4
CPT1A	-0.51	0.11	-1.2
KLF13	0.33	0.08	-2.8
RFTN1	0.00	0.99	∞

1. The effect size of the association of the percentage of neutrophils in blood with methylation in 44 unrelated individuals from the control population in the Leiden Longevity Study.
2. Two-sided p-value for the association between DNA methylation and the percentage of neutrophils in blood.
3. The size of the change in neutrophil percentage in blood, expressed in standard deviations (SD), required to explain in full the association between DNA methylation and prenatal famine exposure (3.3% change in neutrophils is 1 SD change).

Only CDH23 is affected by blood cell heterogeneity, but the change in blood cell composition to explain the observed famine association is so large (~2SD) that this is a highly unlikely explanation.

Supplementary Table 8. Primers to generate the P-DMR CPT1A and INSR constructs

**P-DMR**	**Type**	**Primer: 5' – 3'**	**Restriction enzyme used**
INSR	Forward	TTTGGTGGAGTCTTGCTCTG	
	Reverse	TTACAGACCTGAGCCACTGC	
	Nested forward	ATCAGCTGCAGGAGTGCAATGGCGTGATCT	PstI
	Nested reverse	ATCAGACTAGTGGGCCCATTTCCTTCTTTAG	SpeI
CPT1A	Forward	TGGGTAAGATTGGCAAGGAG	
	Reverse	AATGTGCAGGGATGTGTCTG	
	Nested forward	ATCAGCCTGCAGGTTTGAGACGGGGTCTTGC	SbfI
	Nested reverse	ATCAGACTAGTTGCTGCTTATCACCTTCTCG	SpeI

Supplementary Table 9. Bisulfite primers to check DNA methylation of inserted P-DMR in CpGL-P-DMR/EF1 vector

Primer name	***Primer: 5' -3'***
BS-pCpGL-INSR/EF1 forward	GAGTGATATTATTGGTGGTAGTTTTTA
BS-pCpGL-INSR/EF1 reverse	CTATCCCAACTCTAAACTCATATACTATAT
BS-pCpGL-CPT1A/EF1 forward	TTGGGGTGTAGGAAGAATGTTTAGTAG
BS-pCpGL-CPT1A/EF1 reverse	CTCCTCTCCAACCTAACCCTCAAC

Supplementary Table 10. Primers used to sequence vector and check insert

Primer name	***Primer: 5' -3'***	***Use***
pCpGL_Forward	TAAATCTCTTTGTTCAGCTCTCTG	PCR and Sanger sequencing
pCpGL-Empty_Reverse	TCTTGTAGTCTTGTAGGCTCCTC	Used for basic vector
pCpGL-Insert_Reverse	TTTCTTAATATTCTTGGCATCCT	Used when P-DMR was inserted
